# Sex-biased gene expression across mammalian organ development and evolution

**DOI:** 10.1126/science.adf1046

**Published:** 2023-11-03

**Authors:** Leticia Rodríguez-Montes, Svetlana Ovchinnikova, Xuefei Yuan, Tania Studer, Ioannis Sarropoulos, Simon Anders, Henrik Kaessmann, Margarida Cardoso-Moreira

**Affiliations:** 1 Center for Molecular Biology of Heidelberg University (ZMBH), DKFZ-ZMBH Alliance; D-69120 Heidelberg, Germany; 2 BioQuant, Heidelberg University; D-69120 Heidelberg, Germany; 3 Evolutionary Developmental Biology Laboratory, The Francis Crick Institute; London NW1 1AT, UK

## Abstract

Sexually dimorphic traits are common among mammals and are specified during development through the deployment of sex-specific genetic programs. Because little is known about these programs, we investigated them using a resource of gene expression profiles in males and females throughout the development of five organs in five mammals (human, mouse, rat, rabbit and opossum) and a bird (chicken). Sex-biased gene expression varies considerably across organs and species and is often cell type-specific. Sex differences increase abruptly around sexual maturity instead of increasing gradually during organ development. Finally, sex-biased gene expression evolves rapidly at the gene level, with differences between organs in the evolutionary mechanisms used, but slower at the cellular level, with the same cell types being sexually dimorphic across species.

In many vertebrates, sex differences are the most extreme phenotypic variation seen within species (1). While some sexually dimorphic traits are evident to the naked eye (e.g., differences in body size or plumage), many are not visible yet are no less important (e.g., differences in drug clearance or immune responses) (2, 3). Sexually dimorphic traits are specified at different points during development through sex-specific gene expression programs. Males and females are almost identical genetically, only differing in their sex chromosomes (X and Y in mammals, Z and W in birds). Genes on these chromosomes (e.g., *SRY* in mammals or *DMRT1* in birds) initiate the sex-determination pathways responsible for the differentiation of the gonads into ovary or testis (1, 4). Upon sexual differentiation, the gonads start producing sex hormones (e.g., testosterone and estrogens) that reach different parts of the body and bind to their receptors on target cells. The hormonal signals trigger gene regulatory cascades that differ between males and females, leading to differential gene expression between the sexes and the development of sexually dimorphic traits (5). In mammals, the development of most sexual phenotypes depends on sex hormones (6), while in birds, although sex hormones still play a role, sexual phenotypes are largely cell-autonomous, with somatic cells carrying an inherent sex identity (7).

Genes with sexually dimorphic expression are called “sex-biased” genes and include those expressed exclusively in one sex and those expressed in both sexes but with different levels. Sex-biased genes are called male-biased or female-biased, depending on which sex shows the higher expression level. Apart from the sex determination pathways, little is known about sex-specific developmental gene expression programs. Most studies on sex-biased gene expression focused on adults (e.g. (8–10)) when phenotypic sex differences are greatest (11). However, some sexually dimorphic phenotypes are established early in development (12, 13), and it is unclear when the sexual dimorphisms observed in adults first emerge. Therefore, it is critical to study sex differences in a developmental context.

Across animals as diverse as butterflies, flies, and birds, there is a high species turnover of Sex-biased expression (14–16). While the same is likely true for mammals, the reported extent of conservation of sex-biased expression differs between studies (8, 17–19). Understanding the evolution of sex-biased expression within a developmental framework is essential because evolutionary and developmental processes are strongly intertwined, with species differences being usually lower early in organ development (20, 21). Here, we use bulk RNA sequencing (RNA-seq) time series datasets to describe the extent, temporal dynamics, and conservation of sex-biased gene expression across organs and species, and combine these data with single-cell RNA-seq (scRNA-seq) and ChIP-seq datasets to infer the cellular and molecular mechanisms responsible for sexually dimorphic expression and their evolution across mammals.

## Extent of sex-biased expression across organs and species

**Fig. 1:**
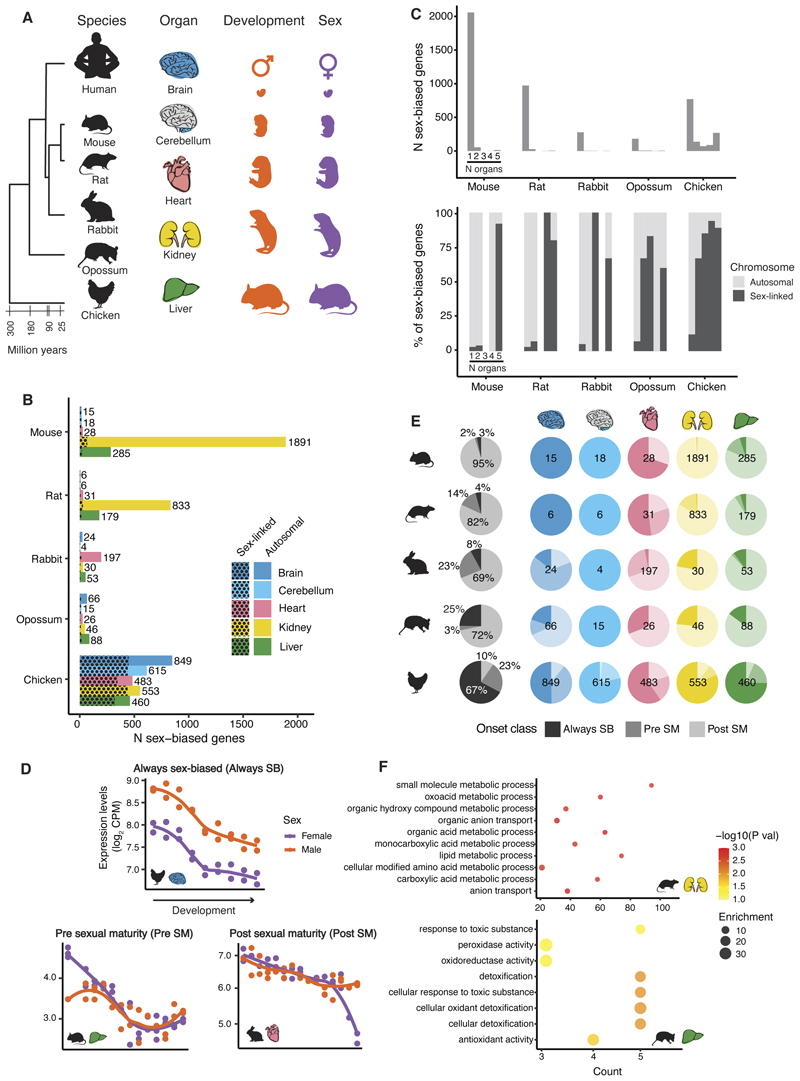
Extent and onset of sex-biased gene expression. **(A)** Summary of the dataset. (B) Number of sex-biased genes by species and organ. Spotted pattern means genes located on sex chromosomes, X/Y in mammals (for rat, rabbit and opossum these only include X-linked genes as Y-linked genes were not present in the assemblies), W/Z in chicken. **(C)** Number of Sex-biased genes and chromosomal location as a function of the number of organs where genes are sex-biased. **(D)** Examples of genes belonging to each of the onset classes, *RPL17* in chicken brain, *Pagr1a* in mouse liver and *LUC7L* in rabbit heart, respectively. CPM = counts per million. **(E)** Percentage of sex-biased genes belonging to each of the onset classes, always sex-biased (Always SB), sex-biased pre sexual maturity (Pre SM) or sex-biased post sexual maturity (Post SM). Depending on the species, 0.001-0.03% of genes were not assigned to any of the 3 categories and are not in the plot (22). Total number of sex-biased genes per organ and species inside each pie plot. **(F)** Enriched biological processes among genes that become sex-biased after sexual maturity in rat kidney and opossum liver (n = 688 in rat and, n = 75 in opossum; Benjamini–Hochberg-adjusted *P* < 0.05, hypergeometric test).

To study sex-biased expression during organ development, we analyzed RNA-seq time series data (20) from six species (human, mouse, rat, rabbit, opossum, and chicken) covering the development of five organs (brain, cerebellum, heart, kidney, and liver) (Fig. 1A). The time series span from early organogenesis to adulthood (7-16 stages) with 1-3 replicates per sex per stage, except for human, where the time series end shortly after birth, often with only one replicate per sex (Table S1). We identified sex-biased genes using an approach that combines information from four time series differential expression algorithms that we validated through extensive simulations (22). Because the human time series end shortly after birth, we adopted a different strategy to call sex-biased expression in humans. We required the set of genes identified as sex-biased during prenatal development also to be classified as sex-biased in adults using the Genotype-Tissue Expression (GTEx) resource (9) (Table S7). Consequently, the set of human sex-biased genes is composed only of genes that differ in adults that started differing between the sexes pre- or perinatally. In contrast, the sets of sex-biased genes for the other species include genes that are differentially expressed at any point during development (22). The gene expression profiles and sex-bias status can be explored interactively (https://apps.kaessmannlab.org/sexbiasapp).

We first examined the levels of sex-biased expression in mouse, rat, rabbit, opossum, and chicken. We found considerable differences between species and organs in the levels of Sex-biased expression. Chicken has the highest percentage of sex-biased genes, with 8% of the genes tested being sex-biased in at least one organ (1337 genes). Across mammals, mouse has the highest percentage of sex-biased genes (2127, 5.9% of all genes tested), followed by rat (1005 genes, 3.9%), with rabbit and opossum showing considerably fewer sex-biased genes (287 and 200 genes, respectively, ~1%). The lower number of sex-biased genes in opossum is at least partly a consequence of the lower sensitivity of our approach in this species (Fig. S1C) (22). Within each species, the number of sex-biased genes varied extensively across organs (Fig. 1B). For example, in mouse, only 15 genes are sex-biased in the brain, whereas 1891 genes are Sex-biased in the kidney. Notably, the organs with the most sexually dimorphic transcriptomes differed between species: kidney in mouse and rat, heart in rabbit, liver in opossum, and brain in chicken (Fig. 1B).

Most genes are sex-biased in a single organ, as previously observed (8–10) (Fig. 1C). However, these genes tend to be expressed in multiple organs, with only a minority of genes sex-biased in one organ (~3-9% across species) being specifically expressed in that organ (organ-specificity index (τ) > 0.8). In mammals, only a few genes are sex-biased across multiple organs, and these are strongly enriched for genes on the sex chromosomes. While genes on sex chromosomes contribute to ~3-7% of organ-specific sex-biased genes (consistent with ~2-10% of genes in each species being on sex chromosomes), they make up ~60-90% of genes sex-biased across all organs (*P <* 0.01 in all species, *X^2^*-test, Fig. 1C). This latter category includes Y-linked genes, long non-coding RNAs involved in X-chromosome inactivation (e.g., *XIST* in placental mammals and *RSX* in opossum), X-linked genes that escape X-chromosome inactivation, and a small number of autosomal genes (e.g., *Uba5* in mouse) (Tables S2-7) (22). In contrast to mammals, in chicken, hundreds of genes are sex-biased across multiple organs. Most of these genes are on the Z chromosome and reflect the lack of a global dosage compensation mechanism (23). Because males have two Z chromosomes and females only one, most Z-linked genes show consistent higher expression in males than in females across multiple organs (461 genes, Fig. S2). In all species, genes that are sex-biased across multiple organs predominantly have the same direction of bias (male or female bias) across organs (from 78% of multi-organ sex-biased genes in rat to 100% in opossum).

## Onset of sex-biased expression

Because sexually dimorphic phenotypes are most noticeable in adults, we expect adults to show the highest levels of sex-biased expression (11). However, it is unkown how much sex-biased expression exists during organ development and when the onset of the sex differences observed in adults occurs. To answer these questions, we determined the onset of sexually dimorphic expression for each sex-biased gene using soft clustering (22, 24) (Tables S8-13). We consistently found three classes of sex-biased genes: 1) genes sex-biased across all developmental stages, 2) genes sex-biased before sexual maturity, and 3) genes that become Sex-biased around or after sexual maturity (Fig. 1D).

In mammals, most genes become sex-biased around or after sexual maturity (69-95% of Sex-biased genes, depending on the species) (Fig. 1E). These genes are enriched among functions specific to each organ (Fig. 1F and Fig. S3B-D), including detoxification in the mouse, rat, and opossum liver and transport of small molecules in the mouse and rat kidney. These enrichments implicate sex-biased genes in the specific physiological processes executed by each organ and may underlie known sex differences in these processes (25–27).

A considerably smaller fraction of genes show differences before sexual maturity (2-23% of all sex-biased genes) (Fig. 1E). Among these are genes that start to differ between the sexes prior to sexual maturity and continue to do so in adults (8 genes in mouse liver, 7 genes in rat heart, 14 genes in rabbit brain and 23 genes in rabbit heart), genes that differ across several time points but are similarly expressed between the sexes in adults (8 genes in opossum brain and 130 genes in rat kidney), and genes that are sex-biased only during early development (31 genes in mouse liver, 8 genes in rat liver, 31 genes in rabbit heart and 1 gene in rabbit liver). Many of the mouse genes that are sex-biased before sexual maturity are associated with sexually dimorphic phenotypes (Table S14). The International Mouse Phenotyping Consortium (IMPC) (28) generated single gene knockout lines for 10 of the 39 mouse genes with early onset of sex-biased expression, and 5 showed sexually dimorphic phenotypes (50% vs 14% of sexually dimorphic phenotypes among a total of 8619 knockouts, *P* < 0.01, *X*^2^-test). A knockout of *Ndrg4*, Sex-biased in the liver, leads to increased levels of circulating creatinine and blood urea nitrogen in females but not in males (28). Similarly, males without a functional *Casq1* (sex-biased in the liver) show abnormal cholesterol homeostasis, whereas females do not (28).

Finally, in mammals, some genes are sex-biased across all developmental stages (3-25% of Sex-biased genes) (Fig. 1E). These genes are also sex-biased across multiple organs and predominantly located on the sex chromosomes (*P* < 0.01, *X*^2^-test). Therefore, there is a set of genes that are sex-linked and sex-biased throughout the entire development of multiple organs. This set includes Y-linked genes, long non-coding RNAs involved in X-chromosome inactivation, and the small number of X gametologs of ubiquitously expressed Y-linked genes (e.g., *EIF2S3X*, *DDX3X*, *KDM6A* or *KDM5C*), which escape X-chromosome inactivation. There are also a handful of autosomal genes (Fig. S3E) that are sex-biased across the entire development of multiple organs, including *Uba5* in mouse, *Ddx3y* in rat (located on chromosome 13), a rabbit ortholog of the human *EIF1AY*, and *ZNF451* and two more genes in opossum.

Additionally, a few genes in each species are sex-biased across all developmental stages in an organ-specific manner (e.g., *Vamp7* in the mouse heart and *Hip1r* in the rat kidney).

In organs with high levels of sex-biased expression (e.g., mouse liver) most genes become Sex-biased around or after sexual maturity, whereas in organs with low levels of sex-biased gene expression (e.g., mouse brain), most genes are always sex-biased (Fig. 1E).

The set of human sex-biased genes consists of genes that start differing between the sexes before or near birth and remain sex-biased in adults (as identified by GTEx, (22)). Among mammals, human has the largest number of genes in this category (78 genes), followed by rabbit (43), mouse and rat (26), and opossum (22), though we may be underpowered for the marsupial (Fig. S3F)(22). These sex-biased genes are uniformly distributed among the organs, and while they are enriched for sex-chromosome genes, many are autosomal (Fig. S3G).

In chicken, the temporal dynamics of sexually dimorphic expression are opposite to those in mammals (Fig. 1E). Only a minority of genes (~10% of all sex-biased genes) become sex-biased around or after sexual maturation. Most genes (~67%) are sex-biased across all developmental stages and across organs. While most of these always sex-biased genes are sex-linked (~85% of always sex-biased genes) and reflect the lack of global dosage compensation, 15% are autosomal.

## Conservation of sex-biased expression

**Fig. 2:**
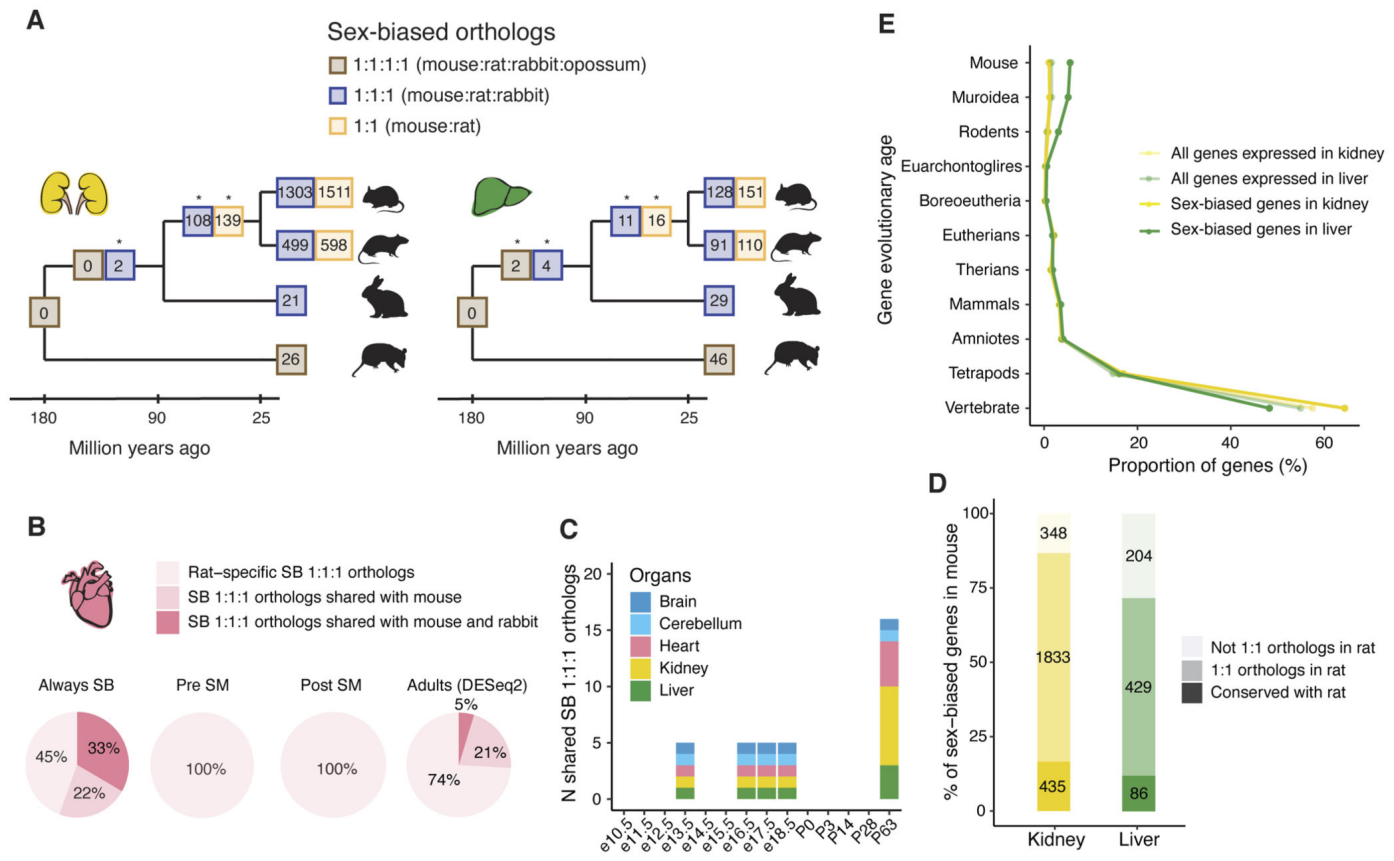
Conservation of sex-biased gene expression. **(A)** Phylogeny showing the number of sex-biased orthologs in kidney and liver across mammals. * Benjamini–Hochberg-adjusted *P* < 0.05, permutation test. The different numbers reflect the different sets of 1:1 orthologs used. For example, the set of 1:1 (mouse:rat) orthologs includes all 1:1:1 (mouse:rat:rabbit) orthologs plus genes that are only 1:1 orthologs between mouse and rat. **(B)** Percentage of sex-biased 1:1:1 orthologs in rat heart that are only sex-biased in rat, sex-biased in rat and mouse or sex-biased in rat, mouse and rabbit, depending on the onset of sex-biased expression. **(C)** Number of shared sex-biased 1:1:1 orthologs in mouse, rat and rabbit at different developmental stages (matched across species) using classical differential expression analysis (DESeq2). **(D)** Number and percentage of sex-biased genes in mouse kidney and liver that are either also sex-biased in rat, have a 1:1 ortholog in rat or do not have a 1:1 ortholog in rat. **(E)** Proportion of expressed and sex-biased genes in the mouse kidney and liver and according to their evolutionary age.

Next, we investigated the extent of conservation of sex-biased expression across species (i.e., mouse, rat, rabbit, opossum and chicken, with comparable time series). We determined the overlap between the sets of sex-biased genes in the different species according to the onset of sex-biased expression (i.e., always sex-biased, sex-biased prior to sexual maturity, or sex-biased post sexual maturity). There are no sex-biased genes conserved between mammals and chicken or across all mammals (Fig. 2A and Fig. S4A). We identified only five sex-biased genes conserved across mouse, rat, and rabbit (Y-linked genes excluded). These include three genes that are always sex-biased (*Xist* and the X gametologs *Eif2s3x* and *Kdm6a*) and two genes Sex-biased in the liver post sexual maturity (*Cux2* and *Nipal1*). Except for *Nipal1*, these genes are also sex-biased in the corresponding organs in adult humans (9), suggesting they are conserved across placental mammals. For mouse, rat, and rabbit, there is little conservation outside the set of genes that are always sex-biased (Fig. 2B, Fig. S4B) (22).

Previous studies in adults (8, 17–19) also found low levels of conservation of sex-biased expression, but the number of conserved sex-biased genes varied considerably between studies, ranging from a handful (17, 18) to several hundred (8, 19). Because our approach for calling Sex-biased genes is underpowered to detect sex differences limited to one or two stages (Fig. S1) (22), which includes adult-only sex differences, we could be underestimating the extent of conservation of sex-biased expression. To test this possibility, we applied classical differential expression analysis using DESeq2 (22, 29) to the adults in our dataset and independently to four prenatal stages. As expected, there is a good overlap between sex-biased genes identified in adults with DESeq2 and those identified by our time series approach (Fig. S4C) (22). The newly identified adult sex-biased genes showed significantly smaller differences in expression levels between the sexes than those identified by our time series approach (*P* < 0.0001, Wilcoxon rank-sum test, Fig. S4D). Using the set of adult sex-biased genes identified by DESeq2, we identified a higher number and proportion of sex-biased genes conserved across mouse, rat and rabbit (17 vs 5 using the time series approach, Fig. 2C and Fig. S4B). In contrast, the analysis of the four prenatal stages identified only *Xist* as conserved across all three species (Fig. 2C). These results confirm that there are few sex-biased genes pre-sexual maturity, that only a handful of them are conserved across species, and that these are better identified with our time series approach. However, they also suggest we are underestimating the extent of sex-biased expression in adults for genes with smaller differences in expression levels between the sexes. To overcome this limitation, we created an extended set of sex-biased genes that combines the time series calls with those made with DESeq2 in adults.

Our analyses indicate that sexually dimorphic expression evolves rapidly, with conservation of sex-biased expression during development restricted to a few key genes, most of which are Sex-biased across all developmental stages and organs. These genes include *Xist*, the small number of X gametologs whose partners on the Y chromosome are ubiquitously expressed, and a few others (Table S15).

## Evolutionary age of sex-biased genes in mouse and rat

To explore the fast evolution of sex-biased expression, we focused on the two most closely-related species, mouse and rat, using the extended set of sex-biased genes (combining the time series and adult-only calls). In both species, the two most sexually dimorphic organs are the kidney and liver, but only a small percentage of genes are sex-biased in both species (17% in kidney, 12% in liver, Fig. 2D). The conserved sex-biased genes tend to have the same direction of sex bias in the two species (64% in kidney and 73% in liver) and are involved in important processes, including transmembrane transport in the kidney and redox reactions in the liver.

Among the genes sex-biased in mouse but not in rat, it is important to distinguish between those that have 1:1 orthologs in rat and those that do not (because of gene duplication or loss). While in the kidney, most mouse sex-biased genes have 1:1 orthologs in the rat (87%), in the liver, 28% of mouse sex-biased genes do not have a 1:1 ortholog in rat. This suggests that many sex-biased genes in the mouse liver duplicated in the mouse and/or rat lineages, or were lost in rat. To further explore these possibilities, we determined when mouse sex-biased genes first appeared during evolution, that is, when they first arose through gene duplication (30). For most genes in the kidney, the sex differences are recent, but the genes themselves are old (Fig. 2E). By contrast, in the liver, many sex-biased genes appeared recently in evolution, with at least 5% being mouse-specific (a likely underestimation because we could not assign an evolutionary age for 10% of sex-biased genes in the liver vs only 5% in the kidney) (22).

Because newly emerged genes are amongst the least studied (31, 32), we manually examined the annotations for these genes and found many derived from the expansion of three gene families, the cytochrome P450 family (20/77 genes), the major urinary protein family (16/77 genes) and the Slc22 transporter family (5/77). All three families have undergone successive gene duplications (33–35) and are involved in critical sex functions. The cytochrome P450 family is involved in the metabolism of xenobiotics and the transformation of endobiotics like steroid hormones, processes with many sex differences (27). The major urinary protein family codes for sex pheromones and is involved in creating scent marks used for male-male competition, female assessment of males, and kin recognition (33). The rodent-specific expansion of the Slc22 transporter family has been associated with the transport of conjugated sex hormones (35). In the rat liver, recently-emerged sex-biased genes also belong to the major urinary protein family (4/37 genes), which has expanded in parallel in mouse and rat (36, 37), and the P450 family (2/37). Although there are few conserved sex-biased orthologs in the mouse and rat liver, there is conservation of sex-biased expression at the level of gene families.

The analysis of the evolutionary age of sex-biased genes uncovered important differences between organs. In the mouse and rat kidney, evolutionarily old genes quickly evolved sex differences in expression. This is also true for the rabbit heart (Fig. S4E), the most sexually dimorphic organ in this species, where most sex-biased genes have 1:1 orthologs in mouse. However, in the liver, the evolution of sex differences has often involved the evolution of new genes through the independent expansion of the same gene families.

## Cellular basis of sex-biased expression

Sex differences in expression at the bulk tissue level can be a consequence of the differential expression of genes in the same cell types between males and females, differences in the proportion of cell types between the sexes, and/or genes being expressed in different cell types between males and females. Distinguishing between these scenarios requires investigating sex differences at the single-cell level. Therefore, we used single-cell datasets to identify the cell population(s) that express the sets of sex-biased genes identified at the bulk tissue level (22).

**Fig. 3:**
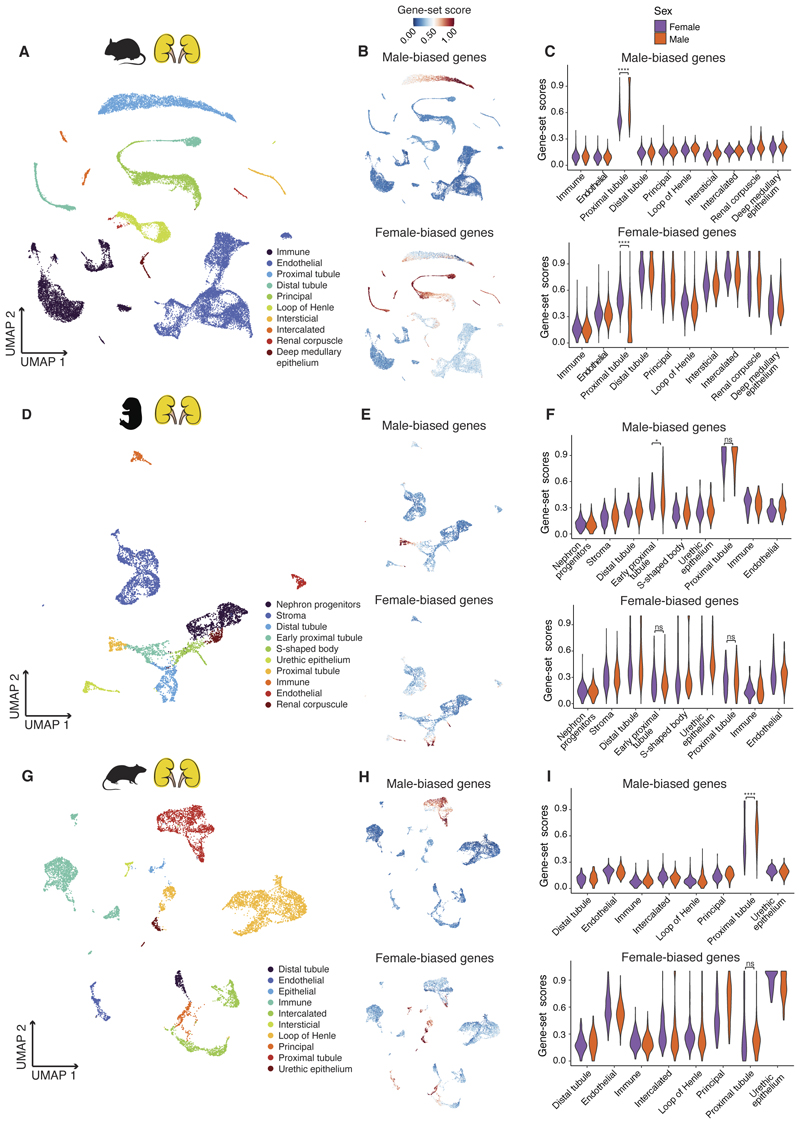
Cellular basis of sex-biased gene expression in mouse and rat kidney. **(A)** UMAP of adult mouse kidney scRNA-seq dataset (data from (38)) (29611 cells). **(B)** UMAPs illustrating expression of male-biased (up) and female-biased (down) genes in adult mouse kidney. **(C)** Distribution of male-bias (up) and female-bias (down) gene-set scores according to cell types and separating by male and female cells in adult mouse kidney (**** Benjamini–Hochberg-adjusted *P* < 0.0001, two-sided Wilcoxon rank-sum test). **(D)** UMAP of prenatal mouse kidney scRNA-seq dataset (data from (43)) (5168 cells). **(E)** UMAPs illustrating expression of male-biased (up) and female-biased (down) genes in prenatal mouse kidney. **(F)** Distribution of male-bias (up) and female-bias (down) gene-set scores according to cell types and separating by male and female cells in prenatal mouse kidney (* Benjamini–Hochberg-adjusted *P <* 0.05, ns means not significant, two-sided Wilcoxon rank-sum test). **(G)** UMAP of adult rat kidney scRNA-seq dataset (data from (45)) (9340 cells). **(H)** UMAPs illustrating expression of male-biased (up) and female-biased (down) genes in adult rat kidney. (I) Distribution of male-bias (up) and female-bias (down) gene-set scores according to cell types and separating by male and female cells in adult rat kidney (**** Benjamini–Hochberg-adjusted *P* < 0.0001, ns means not significant, two-sided Wilcoxon rank-sum test).

**Fig. 4:**
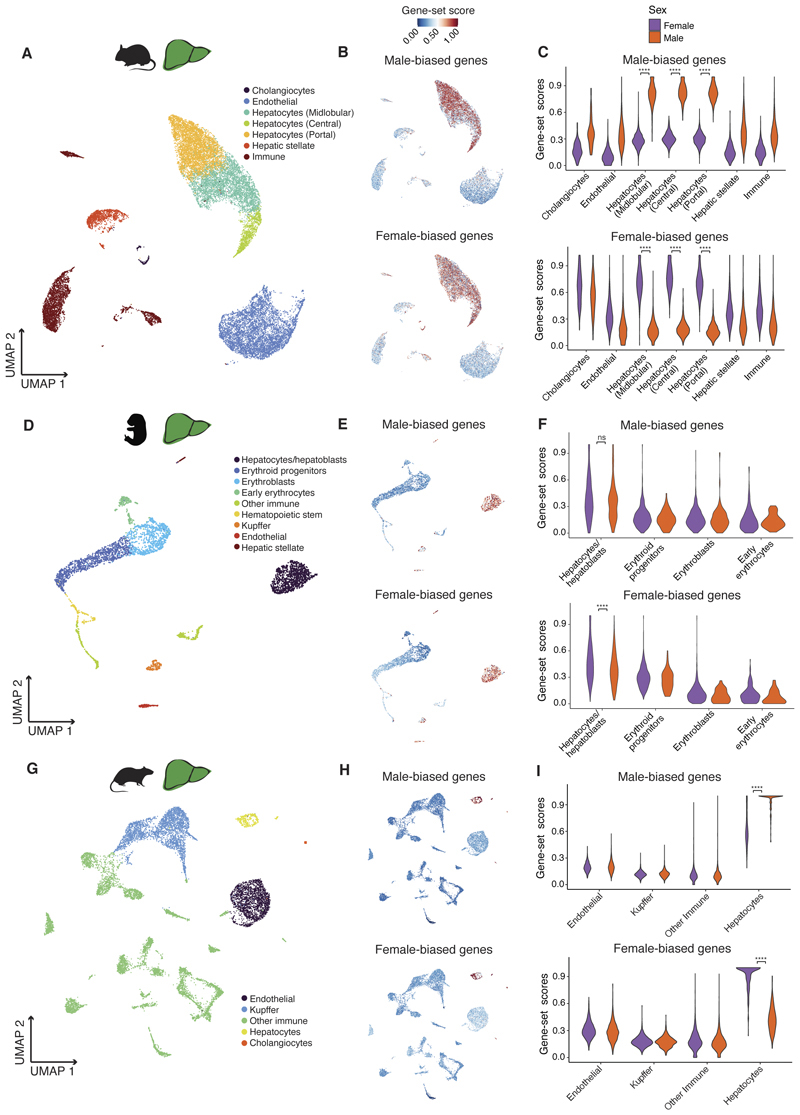
Cellular basis of sex-biased gene expression in mouse and rat liver. **(A)** UMAP of adult mouse liver snRNA-seq dataset (22512 cells). **(B)** UMAPs illustrating expression of male-biased (up) and female-biased (down) genes in adult mouse liver. **(C)** Distribution of male-bias (up) and female-bias (down) gene-set scores according to cell types and separating by male and female cells in adult mouse liver (**** Benjamini–Hochberg-adjusted *P* < 0.0001, two-sided Wilcoxon rank-sum test). **(D)** UMAP of prenatal mouse liver scRNA-seq dataset (data from (44)) (3847 cells). **(E)** UMAPs illustrating expression of male-biased (up) and female-biased (down) genes in prenatal mouse liver. **(F)** Distribution of male-bias (up) and female-bias (down) gene-set scores according to cell types and separating by male and female cells in prenatal mouse liver (**** Benjamini–Hochberg-adjusted *P* < 0.0001, ns means not significant, two-sided Wilcoxon rank-sum test). **(G)** UMAP of adult rat liver scRNA-seq dataset (data from (45)) (11343 cells). **(H)** UMAPs illustrating expression of male-biased (up) and female-biased (down) genes in adult rat liver. **(I)** Distribution of male-bias (up) and female-bias (down) gene-set scores according to cell types and separating by male and female cells in adult rat liver (**** Benjamini–Hochberg-adjusted *P* < 0.0001, two-sided Wilcoxon rank-sum test).

We focused on the two most sexually dimorphic organs in mouse: the kidney and liver. We generated a single-nucleus RNA-seq (snRNA-seq) dataset for four adult mouse livers (22) and used an existing scRNA-seq dataset for the adult mouse kidney (38). We then determined the expression of the extended set of sex-biased genes (combining the time series and adult-only calls) in the single-cell datasets (22). In the mouse liver, male- and female-biased genes are specifically expressed in hepatocytes (Fig. 4A-B), as previously observed (39). Male-biased genes are more highly expressed in male hepatocytes, and female-biased genes are more highly expressed in female hepatocytes (Fig. 4C). In contrast, in the mouse kidney, the cell-type specificity of sex-biased genes differs between male-biased and female-biased genes. Male-biased genes are expressed specifically in the proximal tubule cells, while female-biased genes are not cell-type specific and are expressed across several cell types in addition to the proximal tubule cells (Fig. 3A-B). Despite this difference in cell-type specificity, the expression differences between the sexes are mostly restricted to the proximal tubule cells, as previously suggested (38). In this cell population, male cells express male-biased genes at higher levels than female cells, which in turn express more highly female-biased genes than male cells (Fig. 3C).

Studies suggested small morphological differences between males and females in these two organs, which could reflect cell composition differences (40–42). However, there is no evidence yet for differences in cell type proportions between the sexes, and because of the levels of technical variability in the single-cell datasets, we cannot reliably address this question with these data (Fig. S5A). While we cannot discard the possibility that there are also differences in the abundance of proximal tubule cells in the kidney and hepatocytes in the liver between males and females, our data supports that most sex differences in these two organs are the result of there being a female-version and a male-version of these two cell types and hence, male- and female-biased genes being differentially expressed between the sexes in the same cell types.

In the mouse kidney and liver, most sex-biased genes are only sex-biased starting around puberty (Fig. 1E), and so we do not expect sexually dimorphic expression of these genes before birth. However, we wanted to know where these genes are expressed before showing sex differences. Therefore, we re-analyzed prenatal scRNA-seq datasets from the mouse kidney and liver (43, 44). In the kidney, male-biased genes are already specifically expressed in the proximal tubule cells prenatally, whereas female-biased genes are more broadly expressed (as observed in adults). As expected, before birth, male and female cells express sex-biased genes at similar levels (or with considerably smaller differences than those observed in adults) (Fig. 3D-F). A similar pattern is observed in the prenatal mouse liver. Before birth, male- and female-biased genes are expressed specifically in hepatocytes but with only minor differences in gene expression between male and female cells (Fig. 4D-F). These results show that sex-biased genes are expressed in the same cell types prenatally as in adults and that only after puberty do the cells from males and females start to diverge in their expression.

## Conservation of the sexually dimorphic cell types

Next, we asked if the cell-type specificity of sex-biased expression is conserved across species and, if so, if sex-biased expression is associated with the same cell types across species. To this end, we reanalyzed a scRNA-seq dataset for the rat kidney and liver (45). When we assessed the cell-type specificity of the extended set of rat sex-biased genes in the two organs, we identified the same two cell types we had found in mouse. Male-biased genes are specific to the proximal-tubule cells in the kidney (Fig. 3G-I), and male- and female-biased genes are specific to hepatocytes in the liver (Fig. 4G-I). In both organs, the expression differences between male and female cells were the same as in mouse. These observations are not driven by the set of Sex-biased genes common to both species (Fig. S5B-C). Our results suggest that although sex-biased expression evolves fast at the gene level, it evolves more slowly at the cell type level. This is consistent with a single-cell study of the human kidney (46) that also identified the proximal tubule cells as being sexually dimorphic and driving most sex dimorphisms, despite there being very little conservation in the set of sex-biased genes between rodents and humans.

To understand how sex-biased expression evolved so quickly between mouse and rat, we focused on genes that are sex-biased in only one of the species and determined their expression in the other (where they are not sex-biased). We asked if these genes are expressed in the same cell types in mouse and rat despite the difference in their sex-biased status. In the kidney, we found this to be true. Rat-only male-biased genes are also specifically expressed in the proximal tubule cells in the mouse dataset and vice versa (Fig. S5D-E). In the liver, while mouse-only sex-biased genes are also specifically expressed in hepatocytes in rat, rat-only sex-biased genes are not as hepatocyte-specific as in mouse (Fig. S5F-G). These results indicate a difference both in the sex- and in the cell type-specific regulation of these genes between species.

## Molecular basis of developmental sex-biased expression

Hormones play a critical role in sex-biased expression by differentially activating transcription factors (TFs) and their downstream targets in each sex (47). While sex-biased TFs underlie a large fraction of sex-biased genes (19, 48), sex-biased expression can also be achieved through non-sex-biased TFs, when, for example, differences in hormone concentrations lead to differential rates of TF translocation to the nucleus and transcriptional activation of downstream targets between the sexes (49). To identify TFs responsible for the sex-biased expression in the mouse kidney and liver, we analysed available ChIP-seq datasets for TFs responsive to the growth hormone (key driver of sex differences in the liver (49, 50)), sex-related hormones (androgens and estrogens), and for TFs we classified as sex-biased (22).

**Fig. 5:**
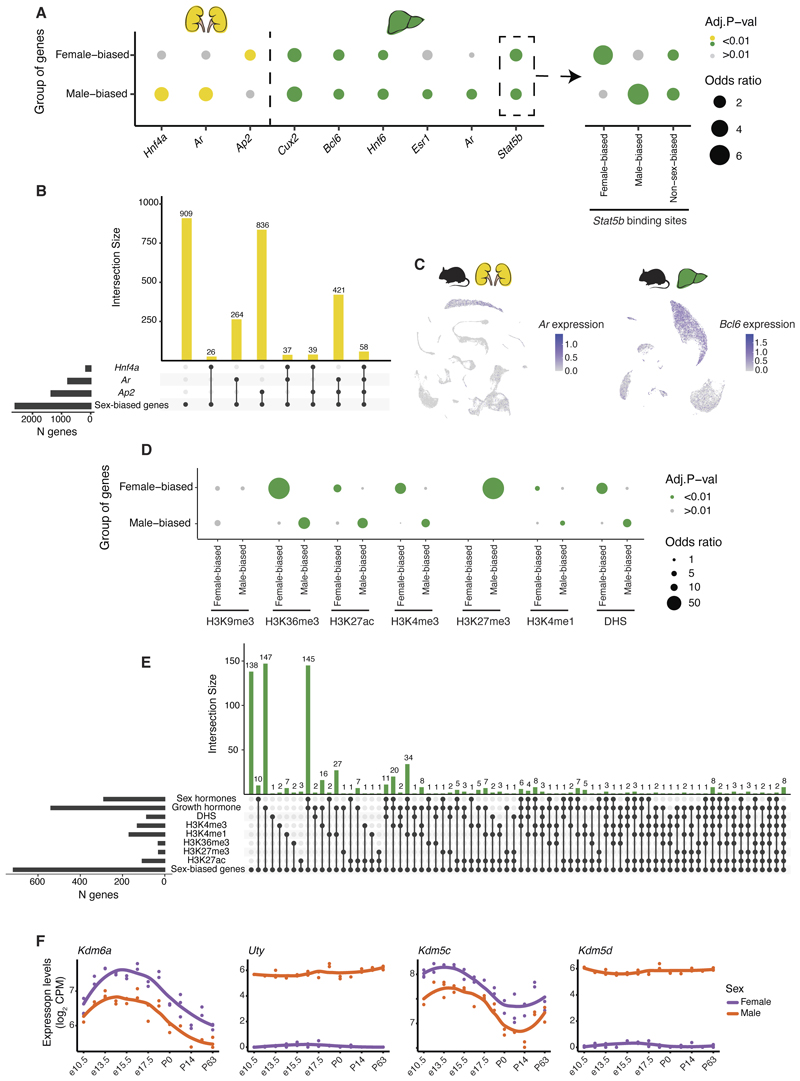
Molecular basis of sex-biased gene expression. **(A)** Enrichment of sex-biased genes for genes regulated by hormone-responsive or sex-biased transcription factors in mouse kidney and liver. **(B)** Number of sex-biased genes targeted by hormone-responsive or sex-biased transcription factors in mouse kidney. **(C)** Examples of cell type-specific expression of 2 transcription factors (*Ar* in proximal tubule cells and *Bcl6* in hepatocytes). **(D)** Enrichment of sex-biased genes for genes located in close proximity to regions with sex differential distribution of epigenetic marks or sex-biased DHS sites. **(E)** Number of sex-biased genes targeted by hormone-responsive or sex-biased transcription factors or in close proximity to regions with sex differential distribution of epigenetic marks or sex-biased DHS sites in mouse liver. **(F)** Gene expression time-courses of *Kdm6a/Uty* and *Kdm5c/Kdm5d* gametologs in mouse liver. CPM = counts per million.

In the mouse kidney, male-biased genes are enriched among the targets of the androgen receptor (*Ar*) and a male-biased TF, *Hnf4a*, known to interact with *Ar* (Fig. 5A). *Ar* and *Hnf4a* are specifically expressed in the proximal tubule cells (Fig. 5C). By contrast, female-biased genes are enriched among the targets of *Ap-2*, a female-biased TF. In the kidney, 65% of sex-biased genes are targeted by at least one of these three TFs (compared to 20% of all genes expressed in the kidney, *P* < 0.01, *X^2^*-test, Fig. 5B).

In the mouse liver, sex differences are mainly driven by the different temporal secretion patterns of the pituitary growth hormone, which is secreted continuously in females and in regular pulses in males (50–53). Both male- and female-biased genes in the mouse liver are enriched for known growth-hormone-related TF targets, including *Stat5b*, a non-sex-biased TF (54), *Bcl6*, a male-biased transcriptional repressor (55,56), *Cux2*, a female-biased repressor (57), and *Hnf6*, another non-sex-biased TF (58) (Fig. 5A). TFs responsive to hormones can act as both inducers and repressors of gene expression depending on their interaction partners (59–62). Previous work showed that only 24% of *Stat5b* binding sites are differentially bound by *Stat5b* in males and females (55). Accordingly, we found that male-biased genes are enriched for targets of male-enriched *Stat5b* binding sites, whereas female-biased genes are enriched for targets of female- enriched *Stat5b* binding sites (Fig. 5A). At the single-cell level, all of these TFs except for *Stat5b* are hepatocyte-specific (Fig. 5C), as previously reported (39), and so are their targets (including those of *Stat5b*).

In the liver, male-biased genes are also enriched among the targets of sex-hormone-responsive TFs, namely *Esr1* and *Ar* (Fig. 5A), which supports that sex hormones drive sex differences in the liver in addition to the growth hormone (63). However, while many sex-biased genes are targeted by both sets of hormones (Fig. 5E), only a few are targeted exclusively by sex-hormone-responsive TFs, supporting a more prominent role in sexual dimorphism for the growth hormone.

To investigate the molecular basis of the fast evolution of sex-biased expression between mouse and rat, we looked at the distribution of binding sites for the TFs driving sex differences in the kidney and liver for genes that are sex-biased only in rat (i.e., we examined their mouse orthologs). In the kidney, the mouse orthologs of rat-only female-biased genes are not enriched among the targets of *Ap-2*, in agreement with their non-sex-biased status (Fig. S6A). However, the mouse orthologs of rat-only male-biased genes are still enriched among the targets of *Ar* and *Hnf4a*, despite not being sex-biased. The lack of male-biased expression in the mouse genes is confirmed by the single-cell datasets and so is unlikely to be a false negative (Fig. S6C-D). This result suggests that another element (e.g., another TF) is also necessary for male-biased expression and that it is its absence that drives the species difference. Alternatively, there could be quantitative differences in the binding of *Ar* and *Hnf4a* that explain the species difference.

In the liver, the mouse orthologs of rat-only female-biased genes differ from mouse female-biased genes by not being enriched among the targets of *Cux2* and the female-biased *Stat5b* binding sites (Fig. S6A), the two TFs that sit atop of the cascade leading to female-biased expression. The mouse orthologs of rat-only male-biased genes differ from mouse male-biased genes by not being enriched among the targets of *Cux2* and *Hnf6*, which work downstream of the growth hormone, and by not being enriched among the targets of sex hormones (i.e., *Esr1*, and *Ar*). The cross-species comparisons for the liver and kidney support a model in which sex-biased expression is the result of a combinatorial process involving multiple TFs, and that sex-biased expression can evolve quickly through the gain/loss of binding sites for a subset of the intervening TFs.

TF binding is associated with chromatin accessibility. Previous studies identified chromatin regions with different accessibility between the livers of male and female mice (64), which are related to different abundances and distributions of epigenetic marks between the sexes (65). We re-examined these datasets and found that sex-biased DNase-hypersensitive sites (DHS) are associated with sex-biased genes (Fig. 5D). We also found that the active chromatin marks H3K4me1, H3K4me3, H3K27ac, and H3K36me3 are associated with male-biased genes in males and female-biased genes in females while the repressive mark H3K27me3 is associated with female-biased genes in males (Fig. 5D). In total, 81% of sex-biased genes in the mouse liver are targeted by at least one of the growth hormone-related TFs, sex-biased DNase-hypersensitive sites or sex-biased chromatin marks (compared to 58% of all genes expressed in the liver, *P* <0.01 *X^2^*-test, Fig. 5E).

The repressive mark H3K27me3 is introduced by the histone-modifying enzymes EZH1/2 (66) and removed by KDM6B and the gametologs *UTY* (male-biased) and *KDM6A* (female-biased) (Fig. 5F). Similarly, the activating mark H3K4me3 is introduced by SETD1a/SETD1b, MLL1/MLL2, and PRDM9 (66) and removed by KDM5A, KDM5B, and the gametologs *KDM5D* (male-biased) and *KDM5C* (female-biased). These gametolog pairs are among the rare genes that show conserved sex-biased expression across placental mammals. Perhaps despite the poor conservation of sex-biased genes across these species, sex differences in expression could ultimately involve similar molecular processes across placental mammals.

## Discussion

We found that sex-biased expression varies dramatically across species, organs, and developmental stages, and that it is often cell-type specific. In mammals, sex-biased expression is rare during organ development. In sexually dimorphic organs, sex-biased expression abruptly increases around sexual maturity. We expected to find a large increase in the number of Sex-biased genes with the onset of sexual maturity, as observed across species as diverse as frogs (67), stick insects (68), and human (69). However, we were surprised by the low levels of Sex-biased expression during the development of organs with strong sex differences in adults. This suggests that in mammals, most sex differences only start at sexual maturity when they are most visible. However, it is possible that some sex differences start before sexual maturity but are not reflected in sex-biased expression. There is evidence for this in our work, for example, in the TFs identified as driving sex-biased expression that are not themselves sex-biased. In striking contrast to mammals, in chicken, most genes are sex-biased across all developmental stages, and most are Z-linked. The lack of a global transcriptional dosage compensation mechanism on the Z chromosome means that ~5% of chicken genes are always differentially expressed between the sexes, irrespective of the organ or developmental stage. This high level of sex-biased expression could underlie the dominance of cell-autonomous processes in driving sexual dimorphisms in birds.

In all species, a small set of sex-linked genes are sex-biased throughout the development of multiple organs. This set contains most of the few genes with conserved sex-biased expression across placental mammals and includes the long non-coding RNAs involved in X chromosome inactivation, ubiquitously expressed Y genes and their X gametologs. In opossum, this set of sex-linked and always sex-biased genes is of special interest because they are prime candidates for underlying sex differences that occur prior to the differentiation of the gonads. Unlike in placental mammals, in marsupials, the development of some secondary sexual traits, like the mammary gland and the scrotum, is independent of hormones (70). The development of these sexual traits depends instead on the number of X chromosomes (71), presumably a dosage difference in a hitherto unknown X-linked gene (72, 73). The small set of X-linked genes that are consistently sex-biased across organs and developmental stages are prime candidates (listed in Table S16). These genes are not sex-biased in placental mammals and most start showing sex differences very early in opossum development, prior to the differentiation of the bipotential gonad (Fig. S7). Two genes, *PHF6* and *DKC1*, are especially promising because, in human, they have been implicated in the development of the urogenital tract (74, 75).

Work across various taxa suggests sex-biased expression evolves fast across species (8, 14–16). Our work strongly supports this observation. Genes that are sex-biased in one species are typically not sex-biased in another, even among closely-related species. However, our work shows that behind this general observation of fast evolution there are key differences in the evolutionary mechanisms used among organs. In the rabbit heart or the mouse and rat kidneys, evolutionarily old genes quickly evolve sex-biased expression through gains of sex-specific regulatory sequences. However, in the mouse and rat livers, newly evolved genes are the drivers of sex differences, with species differences arising through the deployment of species-specific genes. We note, however, that although these genes are species-specific, they are members of gene families with sexually dimorphic members in multiple species. This suggests more conservation across species at the gene family level.

The rapid turnover of sex-biased expression across species could result from nonadaptive genetic drift, or changes in patterns of natural or sexual selection. While our study was not designed to estimate the contributions of these factors directly, some key results implicate changes in natural and sexual selection as drivers of species differences. First, although we observed rapid changes in the identity of the genes that are sex-biased across species, we found sex differences to be limited to specific cell types, and those cell types to be conserved across species in their sexual dimorphism. Genetic drift cannot easily account for these observations. Instead it is more likely that some cells are a hotspot for sexually antagonistic traits because of their functions (e.g., the uptake and secretion of drugs and xenobiotics that affect the sexes differently). Natural selection can resolve intralocus sexual conflicts – which occurs when genes have different expression optima in males and females (76, 77) – through the evolution of sex-biased expression. Second, several sex-biased genes, particularly in the liver, are known to mediate reproductive competition, which is highly suggestive of sexual selection (14). Several liver sex-biased genes belong to gene families involved in reproduction and mate choice, including the major urinary proteins, which encode for pheromones, and the cytochrome P450 family.

The exception to the fast evolution of sex-biased expression is the small number of genes in placental mammals that are always sex-biased. Despite their small number, these conserved Sex-biased genes could be playing important sex-related roles across species, as seems to be the case for the pairs of gametologs *KDM6A/UTY* and *KDM5C/KDM5D*. These pairs code for demethylases responsible for the removal of epigenetic marks that have different distributions in males and females and are involved in the regulation of expression of sex-biased genes in mouse and human (9, 65, 78, 81). This suggests that, in placental mammals, the genes that consistently show differences between the sexes during development could be involved in triggering and/or maintaining sex-specific developmental programs in each sex similarly across species.

## Materials and methods summary

Detailed information on materials and methods are available as supplementary materials (22). We used four time series differential expression algorithms to identify sex-biased genes across organ development: splineTimeR (82), DESeq2 (29), MaSigPro (83), and our own algorithm (22). The sets of sex-biased genes comprised genes called as sex-biased by at least two different time series algorithms, as this approach yielded the best results in terms of sensitivity and specificity as shown by our extensive simulations (done with seqgendiff (22, 84)). We used soft clustering as implemented in GPClust (24) to cluster genes in each organ and species according to their temporal profiles and determine the onset of sex-biased expression.

We generated the mouse liver snRNA-seq dataset from four snap-frozen liver samples that were used for nuclei isolation and single-cell library construction using the Chromium Single Cell RNA Reagent kits (10x Genomics). We processed the sequencing data using cellranger (85).

Publicly available mouse and rat scRNA-seq datasets were obtained from the respective studies (38, 43–45). All single-cell datasets were analyzed with Seurat (87), including quality control, dimensionality reduction, clustering and cell-type annotation. We used ChIPseeker (88) to study TFs and epigenetic marks associated with sex-biased expression in publicly available datasets (48, 55, 58, 64–65, 88–90).

## Supplementary Material

Supplementary Material

## Data Availability

The raw and processed data generated in this study are deposited in ArrayExpress with the accession code E-MTAB-12180. All other data are provided in the manuscript or in (22). The code used to analyze the data is available at https://github.com/Leticia314/Sex_bias_manuscript and also archived at (91). Processed data can be interactively explored at https://apps.kaessmannlab.org/sexbiasapp.

## One-Sentence Summary

Sex differences in gene expression across organ development, cell types, and mammalian evolution.
