# Supplementary material for: Sex-biased gene expression across mammalian organ development and evolution
Source: Science. Author manuscript; Available in PMC 2023 Nov 16. (PMC7615307; doi:10.1126/science.adf1046)
Supplement: Supplementary Material [file EMS190637-supplement-Supplementary_Material.pdf]

Science
AAAS

Supplementary Materials for

Sex-biased gene expression across mammalian organ development and evolution

Leticia Rodríguez-Montes, Svetlana Ovchinnikova, Xuefei Yuan, Tania Studer, Ioannis Sarropoulos, Simon Anders, Henrik Kaessmann and Margarida Cardoso-Moreira

Corresponding authors: l.montes@zmbh.uni-heidelberg.de, h.kaessmann@zmbh.uni-heidelberg.de, margarida.cardosomoreira@crick.ac.uk

The PDF file includes:

Materials and Methods
Figs. S1 to S9
Caption for Tables S1 to S16

Other Supplementary Materials for this manuscript include the following:

Tables S1 to S16 (.xlsx)

Materials and Methods

Detecting sex-biased gene expression

The RNA-seq libraries that comprise the developmental time series for the different organs and species are of similar RNA quality, were sequenced to similar read depths, and show high correlations among replicates, as previously described (*20*). The details on the number of replicates available per sex, organ, and species are provided in table S1.

For all species, we used four time series differential expression algorithms to detect genes that differ between the sexes at some point in development: splineTimeR (*82*), DESeq2 (*29*), MaSigPro (*83*) and our own sex bias detection algorithm (below). These tools work by fitting regression models to two groups and determining whether the models are statistically consistent using a hypothesis test. However, they differ in the regression model and the statistical test they apply, making some algorithms better at detecting specific classes of differentially expressed genes (e.g., genes that differ between the sexes consistently throughout development vs only at certain stages). Because no tool performs optimally for all classes of gene expression differences and to minimize the number of false positives, we only included genes classified as "sex-biased" by at least two pipelines in our final set of sex-biased genes (tables S2-7).

Prefiltering

Before applying each pipeline, we filtered out genes expressed (log2 CPM >0) in less than 3 samples in each organ.

SplineTimeR

As input for splineTimeR (v1.1.0) (*82*), we used normalized log2 transformed counts. This tool fits natural cubic spline curves to time-course data and applies empirical Bayes moderate F-statistics on the coefficients of the spline regression model between two groups for detecting differentially expressed genes over time. We used SplineDiffExprs() with intercept=TRUE and df=3 for detecting sex-biased genes. P-values were adjusted for multiple testing using the Benjamini-Hochberg (BH) procedure (*94*), with an adjusted $P < 0.05$ indicating significance (tables S8-13).

DESeq2 for time-series data

As input for DESeq2 (v1.24.0) (*29*), we used raw counts. DESeq2 is based on a negative binomial model and is a gold standard for pairwise differential expression analysis. To apply it to time course data, we performed a likelihood ratio test (LRT) (*29*) that compares how well a gene's count data fit a "full model" (in this case ~sex + time + sex:time) compared to a "reduced model" (in this case ~ time). P-values were adjusted for multiple testing using the BH procedure (*94*), with an adjusted $P < 0.05$ indicating significance (tables S8-13).

MaSigPro

As input for maSigPro (v1.56.0) (*83*), we used normalized counts. MaSigPro models count data with a negative binomial distribution and performs polynomial regressions to model time-course expression values and a log-likelihood ratio test to detect differentially expressed genes over time. We set degree to 2 in the design matrix (make.design.matrix()), we used p.vector() with counts=TRUE followed by T.fit() with alfa = 0.05, and get.siggenes() with rsq = 0.7 for detecting sex-biased genes. We filtered out genes classified as "influential genes", in which a

few data points are substantially influential to the regression model and are, therefore, potential outliers. P-values were adjusted for multiple testing using the BH procedure (*94*), with an adjusted $P < 0.05$ indicating significance (tables S8-13).

In-house pipeline

To measure differences in gene expression between the sexes, we fitted expression trajectories for males and females with local regression using the locfit package (v1.5-9.1) (*95*). Thus, we obtained smoothed expression values for males and females for every time point. These values were then used to calculate a sex-bias score:

$$SB_{score} = \sqrt{\frac{\max_{n}(m_i - f_i)}{n} \sum_{i}^{n} (m_i - f_i)}$$

where n is the number of time points and m_i, f_i are smoothed expression values at i-th timepoint for males and females, respectively. The maximum difference was selected only among differences in the same direction as the mean value. The score was then multiplied by the sign of the mean difference.

To call a gene sex-biased, the score has to be significantly larger than 0.1. The significance was estimated by a bootstrap-like procedure. For each resampling, a local regression model was refitted, now with weights drawn from a gamma distribution with shape and scale parameters both set to 1. This allowed us to sample points continuously rather than discretely and, therefore, to avoid large gaps in the timeline where fitting a local regression is impossible. The number of resamplings for each gene was defined dynamically, so that after the adjustment for multiple testing with the BH procedure (*94*) the p-values of at least 0.1 would be reachable. The genes with low or inconsistent scores were excluded from the bootstrapping process early, allowing us to resample the minority of genes with potentially sex-biased expression patterns enough times. Genes with adjusted $P < 0.05$ were classified as sex-biased (tables S8-13).

Since the time points were not always evenly sampled and to allow the local regression mode more space to adjust to changes in regions where gene expression changes strongly, we also adjusted the timeline before calculating the scores. To this end, we calculated differences in mean expression between all neighboring time points for each gene, and made the distances between the time points proportional to the median values of those differences. The birth point was set to 0, and the distance to the next available time point was set to 1. These adjusted time point values were then used as X-axis for the local regression models. The bandwidth was defined separately for each tissue and species to always cover 2-3 time points.

DESeq2 for adult data

As input for DESeq2 (*29*), we used raw counts. We applied differential expression analysis with default settings to male and female samples of adult stages in mouse, rat and rabbit (P63, P112, and P186, respectively) separately for each organ. P-values were adjusted for multiple testing using the BH procedure (*94)*, with an adjusted $P < 0.05$ indicating significance (tables S9-11).

Final set of sex-biased genes

To evaluate the performance of the different tools used for identifying sex-biased genes during development, we first generated a simulated dataset (see below) containing 50% of sex-biased and 50% of non-sex-biased genes and used it as an input for the four different tools described above (splineTC, MaSigPro, DESeq2 for time-series data and in-house pipeline). Then we compared the performance of each of the individual tools separately and the performance of taking the overlap of different numbers of tools. This comparison allowed us to quantify the number of true positives, true negatives, false positives and false negatives for each tool and to identify which approach best detects different types of sex-biased genes.

To ensure that our simulated dataset was as similar as possible to our empirical dataset (e.g., same variance across replicates), we selected as a starting point for the simulated dataset the genes from our dataset that had been classified as non-sex-biased by all four tools in each organ and species. To further remove all possible biological sex signal in the data, we additionally shuffled the sex labels at each time point. Next, a known amount of log-fold change signal was added to the male samples of a random sample of genes using the binomial-thinning approach implemented in the R package seqgendiff (v1.2.3) (*84*) using the function 'thin_diff', following a similar approach described in (*96*). The artificial sex signal was added to different numbers of consecutive stages to simulate genes sex-biased in only 1 stage, genes sex-biased at all stages, and all the possibilities in between. For a given number of sex-biased stages, we also considered the position within the time course that those sex-biased stages occupy. Our simulated sex-biased genes represent all possible scenarios of sex bias (i.e., n genes sex-biased at the last stage, n genes sex-biased at the second-to-last stage, etc.). The amount of log-fold change signal added was randomly sampled from the distribution of values of maximum log-fold change per gene for all sex-biased genes in all species.

Our evaluation of the performance of the different tools in isolation and in different combinations (fig. S1) agrees with the findings of Spies and colleagues (*93*). When the methods described above are applied to RNA-seq time series data to identify differential gene expression, most false positives, but not true-positives, are identified by only one of the methods. By running all these tools and selecting the genes that are classified as sex-biased by at least two, we can identify robust differentially expressed genes and avoid most false positives. Therefore, our set of sex-biased genes only includes genes classified as sex-biased by at least two time series pipelines and with a maximal expression in that organ in at least one sex > 1 RPKM.

We also added some lncRNAs known to be involved in sex-related functions to the final set of calls, if they passed the two-pipelines threshold (specifically *Xist* in placental mammals, *RSX* in opossum, and *Jpx* in mouse, rat and human) (tables S2-6). The Venn diagrams with the overlaps of calls from the different methods (after passing the expression threshold) are shown in fig. S8.

In human, our set of sex-biased genes comprises genes classified as "sex-biased" by at least two time series pipelines and classified as sex-biased in adults by Oliva et al. (*9*). Ubiquitously-expressed Y-linked genes that passed the two-pipelines threshold were manually added, as they are male-specific but not included in Oliva et al. (*9*).

For mouse, rat and rabbit, the extended set of sex-biased genes comprises genes classified as "sex-biased" by at least two time series pipelines and genes classified as "sex-biased" in adult samples only by DESeq2 (See above "DESeq2 for adult data").

Over-representation analysis

We performed Gene Ontology enrichment analysis for sex-biased genes using the R package gprofiler2 (v0.2.1) (*97*) and the Gene Ontology library (GO). We used the annotations from each species to do this analysis.

Organ-specificity index

We took the organ-specificity indexes from Cardoso-Moreira et al. (*20*), which are based on the Tau (τ) metric of tissue-specificity (*98*). The index ranges from 0 (broad expression) to 1 (restricted expression). We classified as organ-specific those genes with a $\tau > 0.8$ that showed maximum expression in the same organ where they showed a sex bias.

Onset of sex-biased expression

We used GPClust (*24*), a method to cluster time series using Gaussian processes, to cluster together genes with similar sex-biased temporal behaviour in each organ. As input, we used the fold difference (FD):

$$FD_{i,t} = m_{i,t} - f_{i,t}$$

where for each organ and species $m_{i,t}$ and $f_{i,t}$ denote the log2 median expression levels of gene i at time point t in males and females, respectively. Therefore, for each gene, we had a FD trajectory corresponding to the fold difference between males and females throughout development. GPClust was originally designed to identify different temporal dynamics of single trajectories and not to identify how differences between two temporal trajectories change over time. Consequently, it would cluster genes based on the temporal dynamics of their FDs, regardless of their sign (which is inconvenient as the FD sign indicates male- or female-bias). For example, genes with higher expression in males than in females prenatally but then similar expression levels in adults would cluster together with genes with higher expression in females than in males exclusively in adults, as their fold difference trajectories would be very similar (a straight line with a steep decrease at the end), even though the FD values would be completely different (positive values prenatally and zero values postnatally versus zero values prenatally and negative values postnatally). To overcome this issue, for each gene, at the end of the trajectory of FDs, we prolonged the trajectory by adding as many 0s as time points, which act as a reference point and sets positive and negative FD trajectories apart. If a gene has a trajectory of negative FDs (female bias), at the end the trajectory will go up to 0, and if a gene has a trajectory of positive FDs (male bias), at the end it will go down to 0. This way, GPClust distinguishes between genes that have similar FD trajectories but different FD signs. We set the noise variance (k2.variance.fix) to 0.5 and let GPClust infer the number of clusters. In the few cases where we observed that some genes did not fit well in the assigned cluster, we manually re-assigned them to a different cluster (0.3-7% of the genes depending on the species). Lastly, we grouped all clusters in four classes: 1) sex-biased across all developmental stages (always sex-biased), 2) sex-biased around/after sexual maturation, 3) sex-biased before sexual maturation, or 4) “not assigned” if there was not a clear pattern of sex-biased expression or we suspected they could be

outliers. Only a minority of genes are in this last category (0.001-0.03% depending on the species). The results of the clustering can be found in tables S8-13.

Conservation across species

We used UpSetR (v1.4.0)(*99*) for calculating the overlaps of sex-biased 1:1 orthologs across species. For assessing statistical significance, we performed a permutation test (100 permutations) in which random sets of 1:1 orthologs were sampled in each species, constituting the "simulated" sets of sex-biased 1:1 orthologs (same sizes as the true sets of sex-biased 1:1 orthologs in the corresponding species) and overlaps of the "simulated" sets were computed across species. P-values were adjusted for multiple testing using the BH procedure (*94*), with an adjusted $P < 0.05$ indicating significance. The lists of orthologs between species were obtained using Ensembl's BioMart (*100*) and are based on Ensembl's version 85 annotations.

The low levels of conservation of sex-biased gene expression are robust to lowering the threshold for calling genes sex-biased across species. If we consider genes as sex-biased if classified as such by a single pipeline, only a small number of additional genes shows conservation (*Gas2* across all mammals, and ~30 genes across mouse, rat, and rabbit) (fig. S9A-B and table S15).

We took the evolutionary age of genes from (*20*). These data are described in GenTree (http://gentree.ioz.ac.cn/) (*30*).

Sample collection and ethic statement

We collected liver samples from 9 weeks old adult mice. The use of these samples was approved by an ERC Ethics Screening panel (associated with the ERC Consolidator Grant 615253, OntoTransEvol).

Single-nucleus RNA-seq data production for mouse livers

Liver nuclei were extracted following a published protocol (https://www.nature.com/articles/nprot.2016.015) with small modifications. About 30-50 mg frozen liver was homogenized with a micropestle in 400 µl ice-cold homogenization buffer (250 mM sucrose, 25 mM KCl, 5 mM MgCl2, 10 mM Tris-HCl (pH 8), 0.1% IGEPAL, 1 µM DTT, 0.4 U/µl Murine RNase Inhibitor (New England BioLabs, cat# M0314L), and 0.2 U/µl SUPERas-In (Ambion, cat# AM2694)). The homogenates were triturated gently by a P1000 tip for 10 times, incubated on ice for 5-10 minutes and then centrifuged at 100g for 1 minute at 4 degree to pellet any unlysed tissue chunks. The supernatant was transferred into another 1.5 mL eppendorf tube and centrifuged at 400g for 4 minutes at 4 degree to collect nuclei. The nuclei were washed twice in 400 µl homogenization buffer and strained by a 40 µm Flowmi strainer (Sigma, BAH136800040) during the second wash step to remove nuclei aggregates. The final nuclei pellet was resuspended in 30-50 µl Nuclei buffer (10X Genomics, PN-2000207). To estimate the nuclei concentration, nuclei aliquots were diluted in PBS with Hoechst and PI DNA dyes and counted on Countess II FL Automated Cell Counter (Thermo Fisher Scientific, RRID: SCR_020236). Around 15,000 nuclei were used as input for the single-nuclei RNA-seq experiment. The Chromium Next GEM Single Cell 3′ Reagent Kits v3.1 (PN-1000121, PN-

1000120 and PN-1000213) were used to make single-nuclei RNA-seq libraries. Libraries were quantified on a Qubit Fluorometer (Thermo Fisher Scientific; RRID:SCR_018095) and checked on a Fragment Analyzer (Agilent; RRID: SCR_019417) for quality control. Libraries were sequenced on NextSeq 550 (Illumina; RRID: SCR_016381; 28 cycles for Read 1, 56 cycles for Read 2, 8 cycles for i7 index) to an average depth of (50.85 ± 7.83) thousand reads per nuclei.

Single-nucleus RNA-seq data processing for mouse livers

Raw sequencing data were demultiplexed using cellranger mkfastq (v5.0.1) *(85)*. Then STARsolo (v2.7.9a) (*102*) was used for the initial alignment and Unique Molecular Identifier (UMI) counting with the following parameters (--soloType CB_UMI_Simple –soloUMIlen 12 –readFilesCommand zcat –soloCBwhitelist 3M-february-2018.txt –clipAdapterType CellRanger4 –outFilterScoreMin 20 –soloCBmatchWLtype 1MM_multi_Nbase_pseudocounts –soloUMIfiltering MultiGeneUMI_CR –soloUMIdedup 1MM_CR –soloFeatures Gene GeneFull –soloMultiMappers Unique EM –outSJtype Standard –twopassMode Basic). We enabled counting of multiple-mapping reads using the Expectation-Maximization (EM) algorithm (https://genomebiology.biomedcentral.com/articles/10.1186/s13059-019-1670-y) implemented in STARsolo. Reads were aligned to the GRCm39 genome and Ensembl transcriptome (release 104).

For each sample, we counted the UMI that were mapped to the mature mRNA annotation (exons) and mapped to the pre-mature RNA annotation (exon + intron) separately. We then leveraged the high proportion of intronic UMI counts and total UMI counts that valid barcodes contained to distinguish them from the empty droplets. We used the pre-mature mRNA counts of the valid barcodes for the downstream analyses.

We estimated the doublet score for each barcode using Scrublet (v0.2, python v3.6.8) (*104*) and loaded them together with the gene-barcode count matrix into the Seurat package (v4.1.0) (*86*) for quality control and filtering. Low-quality cells were removed based on their high percentage of mitochondrial reads and low number of detected genes. Putative doublets were identified and filtered by combining high doublet scores, high UMI counts and expression of mutually exclusive markers. After filtering, we normalized and scaled each sample using the SCTransform method (*105*). We then integrated the 4 biological replicates using the canonical correlation analysis (CCA) approach (*106*). The integrated dataset was used for dimensional reduction and clustering. Cell types were annotated based on marker genes provided in the previous liver studies (*107–111*).

Single-cell data analysis

Except for the adult mouse liver, all other datasets were publicly available (*38*, *43–45*). Similar to the pipeline for analyzing the mouse liver snRNA-seq data, we used the Seurat package for filtering, normalization, integration, dimensionality reduction, and clustering. As an initial filtering step to remove low quality cells, we applied the "nFeature_Counts" and mitochondrial gene ratio thresholds recommended by the authors of each study. Doublets were removed based on the doublet score calculated for each barcode with Scrublet (*104*). For each sample, counts were normalized and scaled using regularized negative binomial regression with the SCTtransform() (*105*) function.

We integrated the samples using the IntegrateData() function from Seurat using 3000 anchor features. We used the resulting corrected counts only for UMAP visualization (that typically used the top 30-40 calculated dimensions, depending on the dataset, and a resolution of 0.5) and downstream clustering analysis, and the non-integrated counts for any quantitative comparisons. Cell types were annotated using the markers provided by the authors of the different studies.

The early development opossum single-cell data presented in fig. S7 was retrieved from (*113*). This dataset was generated using full-length RNA sequencing (SMART-Seq v.4), with each cell sequenced as a separate library. Raw sequencing data and cell type annotations (sex, developmental stage) were retrieved from ArrayExpress (E-MTAB-7515) and aligned to the opossum genome (MonDom5) using STAR (v2.7.1a) (*102*). For each cell, we used featureCounts (*114*) to count reads in genes based on an extended annotation of the opossum transcriptome (*115*). Only exonic reads (-t exon) with a minimum mapping quality of 40 (-Q 40) were counted in a strand-specific manner (-s 1). Counts for each cell were combined in a gene-by-cell matrix and normalized for gene length and sequencing depth by calculating Reads Per Kilobase of transcript, per Million mapped reads (RPKM). Expression profiles of sex-biased genes in single cells were summarized by developmental stage and sex (fig. S7).

Assessing the sex of the samples

The mouse prenatal liver dataset (*44*) is a pool of cells from male and female embryos. We classified cells expressing *Xist* (counts > 1) as female and cells expressing at least one Y-chromosome gene (*Uty*, *Eif2s3y*, *Kdm5d*, *Ddx3y*, or *Erdr1*) as male (the few cells expressing both *Xist* and Y-linked genes were discarded).

For assigning the sex of the samples in the other scRNA-seq datasets, we used the sample metadata information provided by the authors.

Gene expression scores

To quantify the expression of a gene set of interest, in our case sex-biased genes, we calculated gene expression scores as described by Sepp et al. (*116*). First, data were normalized by calculating counts per million (CPM) and subsetted for the gene set of interest. Then, we scaled the genes' expression vectors to have a mean of 0 and a variance of 1. We averaged the scaled expression of all genes of interest to compute the score and calculate its 0.01 and 0.99 percentile. Finally, we used the percentiles for capping the score to remove outliers. Values that fell out of these ranges were assigned to the nearest accepted value. We used this approach to assess the distribution among cell populations of all sex-biased genes, male-biased genes (excluding Y-linked genes as they are only present in males) or female-biased genes.

We statistically tested differences in distributions of gene set scores between male and female cells only if there were at least ~50 male and female cells for the corresponding cell type.

TF ChIP-seq data analysis

The ChIP-seq datasets were obtained from the Unibind database (*117*) for *Ap-2*, *Ar* and *Hnf4a* (*88*), for the mouse kidney and *Stat5b*, *Bcl6* (*55*), *Cux2* (*48*), *Hnf6* (*58*) and *Esr1* (*89*) for the

mouse liver. The dataset for mouse liver *Ar* was obtained from (*90*) and peaks were converted from mm8 to mm10 using LiftOver (*118*). For each dataset, we used the annotatePeak() function from the package ChIPseeker (v1.20.0) (*87*) with TxDb.Mmusculus.UCSC.mm10.knownGene (v3.4.7) (*119*) annotation database and default parameters to annotate peaks to the nearest gene. Only peaks at a distance smaller than 10 kb from the transcriptional start site of a gene were considered to be targeting that gene.

To assess the significance of overlaps between sex-biased genes and TF targets, we performed a Fisher's exact test with the testGeneOverlap() function from the GeneOverlap package (v1.20.0) (*120*), and we applied multiple test correction using the Bonferroni procedure (*121*), with adjusted $P < 0.01$ indicating significance.

Histone modification ChIPseq and DHS data analysis

Sex-biased DHS sites were taken from (*64*). Sex-enriched peaks for each chromatin mark were taken from (*65*). The downstream analysis was the same as with the TF ChIP-seq data but using the TxDb.Mmusculus.UCSC.mm9.knownGene (v3.2.2) (*122*) annotation database (the genome version used in those studies).

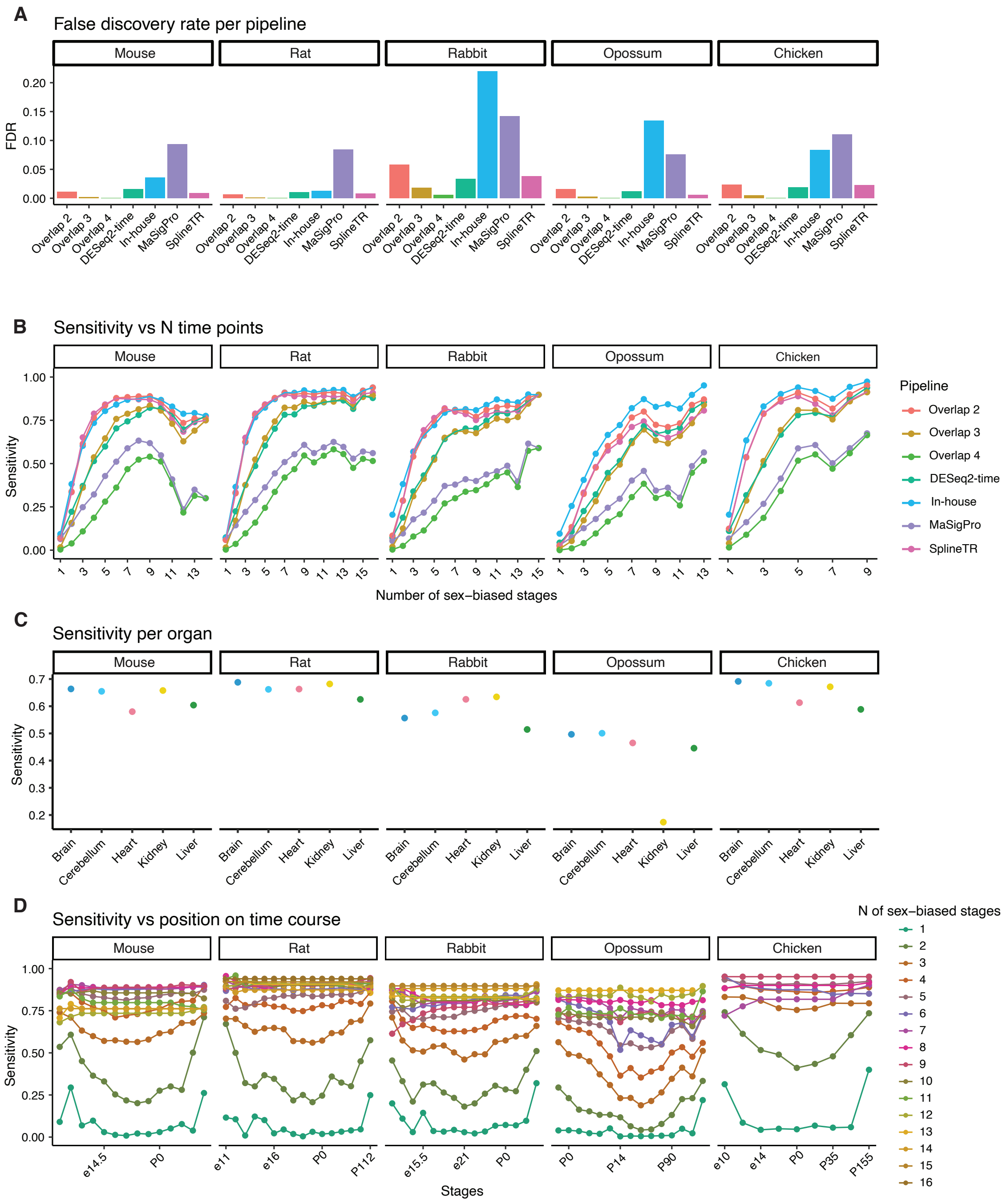

Fig. S1.

(A) False discovery rate (FDR) of every pipeline independently and in combinations for the simulated datasets. **(B)** Sensitivity of the different pipelines for detecting sex-biased genes as a function of the number of sex-biased stages. **(C)** Sensitivity of our approach (calls made by at least two pipelines) across species and organs. **(D)** Sensitivity of our approach as a function of the number of sex-biased stages and their position within the time series.

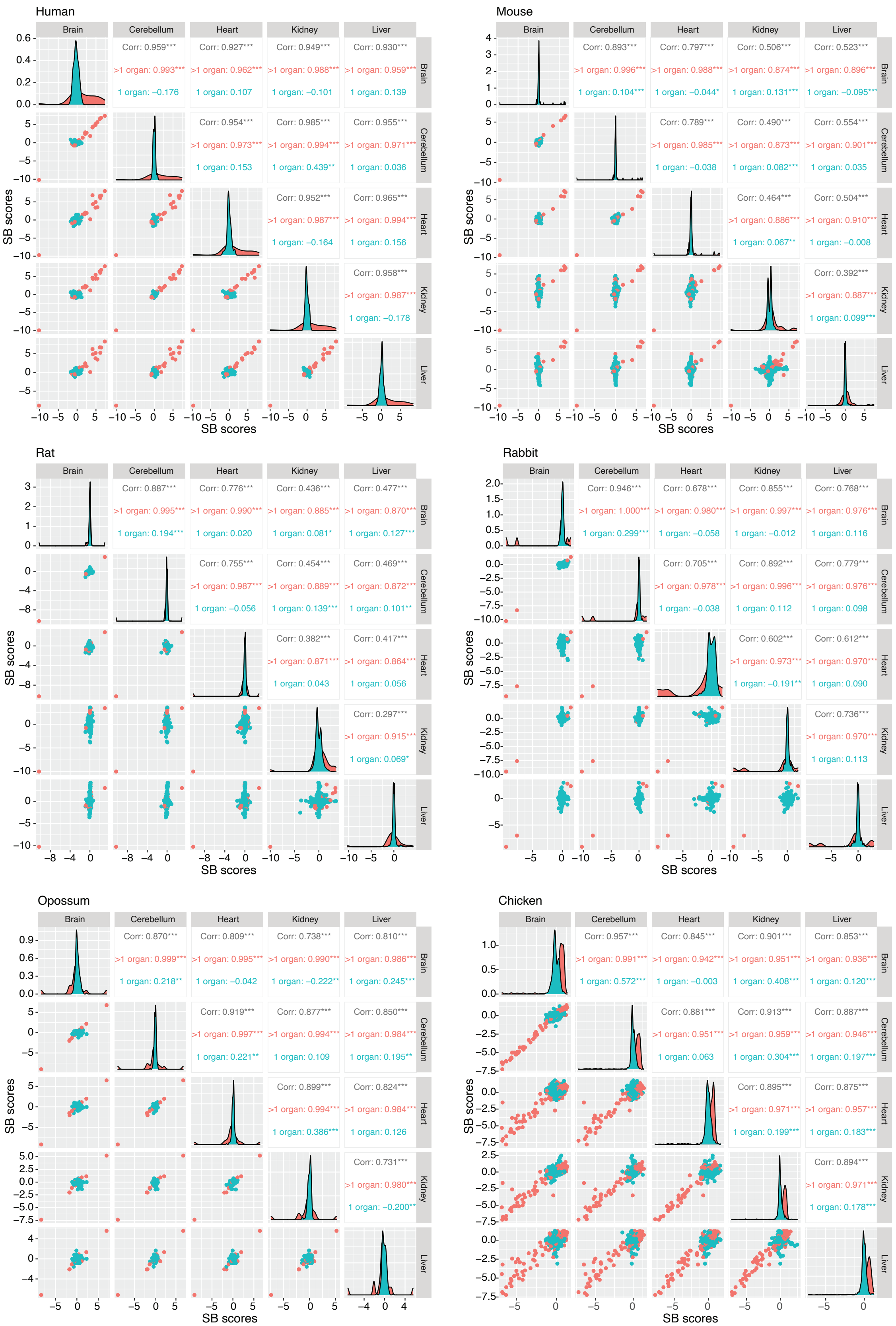
Human
Brain
Cerebellum
Heart
Kidney
Liver
Corr: 0.959***
>1 organ: 0.993***
1 organ: −0.176
Corr: 0.927***
>1 organ: 0.962***
1 organ: 0.107
Corr: 0.949***
>1 organ: 0.988***
1 organ: −0.101
Corr: 0.930***
>1 organ: 0.959***
1 organ: 0.139
Corr: 0.954***
>1 organ: 0.973***
1 organ: 0.153
Corr: 0.985***
>1 organ: 0.994***
1 organ: 0.439**
Corr: 0.955***
>1 organ: 0.971***
1 organ: 0.036
Corr: 0.952***
>1 organ: 0.987***
1 organ: −0.164
Corr: 0.965***
>1 organ: 0.994***
1 organ: 0.156
Corr: 0.958***
>1 organ: 0.987***
1 organ: −0.178
SB scores
SB scores
Mouse
Brain
Cerebellum
Heart
Kidney
Liver
Corr: 0.893***
>1 organ: 0.996***
1 organ: 0.104***
Corr: 0.797***
>1 organ: 0.988***
1 organ: −0.044*
Corr: 0.506***
>1 organ: 0.874***
1 organ: 0.131***
Corr: 0.523***
>1 organ: 0.896***
1 organ: −0.095***
Corr: 0.789***
>1 organ: 0.985***
1 organ: −0.038
Corr: 0.490***
>1 organ: 0.873***
1 organ: 0.082***
Corr: 0.554***
>1 organ: 0.901***
1 organ: 0.035
Corr: 0.464***
>1 organ: 0.886***
1 organ: 0.067**
Corr: 0.504***
>1 organ: 0.910***
1 organ: −0.008
Corr: 0.392***
>1 organ: 0.887***
1 organ: 0.099***
SB scores
SB scores
Rat
Brain
Cerebellum
Heart
Kidney
Liver
Corr: 0.887***
>1 organ: 0.995***
1 organ: 0.194***
Corr: 0.776***
>1 organ: 0.990***
1 organ: 0.020
Corr: 0.436***
>1 organ: 0.885***
1 organ: 0.081*
Corr: 0.477***
>1 organ: 0.870***
1 organ: 0.127***
Corr: 0.755***
>1 organ: 0.987***
1 organ: −0.056
Corr: 0.454***
>1 organ: 0.889***
1 organ: 0.139***
Corr: 0.469***
>1 organ: 0.872***
1 organ: 0.101**
Corr: 0.382***
>1 organ: 0.871***
1 organ: 0.043
Corr: 0.417***
>1 organ: 0.864***
1 organ: 0.056
Corr: 0.297***
>1 organ: 0.915***
1 organ: 0.069*
SB scores
SB scores
Rabbit
Brain
Cerebellum
Heart
Kidney
Liver
Corr: 0.946***
>1 organ: 1.000***
1 organ: 0.299***
Corr: 0.678***
>1 organ: 0.980***
1 organ: −0.058
Corr: 0.855***
>1 organ: 0.997***
1 organ: −0.012
Corr: 0.768***
>1 organ: 0.976***
1 organ: 0.116
Corr: 0.705***
>1 organ: 0.978***
1 organ: −0.038
Corr: 0.892***
>1 organ: 0.996***
1 organ: 0.112
Corr: 0.779***
>1 organ: 0.976***
1 organ: 0.098
Corr: 0.602***
>1 organ: 0.973***
1 organ: −0.191**
Corr: 0.612***
>1 organ: 0.970***
1 organ: 0.090
Corr: 0.736***
>1 organ: 0.970***
1 organ: 0.113
SB scores
SB scores
Opossum
Brain
Cerebellum
Heart
Kidney
Liver
Corr: 0.870***
>1 organ: 0.999***
1 organ: 0.218**
Corr: 0.809***
>1 organ: 0.995***
1 organ: −0.042
Corr: 0.738***
>1 organ: 0.990***
1 organ: −0.222**
Corr: 0.810***
>1 organ: 0.986***
1 organ: 0.245***
Corr: 0.919***
>1 organ: 0.997***
1 organ: 0.221**
Corr: 0.877***
>1 organ: 0.994***
1 organ: 0.109
Corr: 0.850***
>1 organ: 0.984***
1 organ: 0.195**
Corr: 0.899***
>1 organ: 0.994***
1 organ: 0.386***
Corr: 0.824***
>1 organ: 0.984***
1 organ: 0.126
Corr: 0.731***
>1 organ: 0.980***
1 organ: −0.200**
SB scores
SB scores
Chicken
Brain
Cerebellum
Heart
Kidney
Liver
Corr: 0.957***
>1 organ: 0.991***
1 organ: 0.572***
Corr: 0.845***
>1 organ: 0.942***
1 organ: −0.003
Corr: 0.901***
>1 organ: 0.951***
1 organ: 0.408***
Corr: 0.853***
>1 organ: 0.936***
1 organ: 0.120***
Corr: 0.881***
>1 organ: 0.951***
1 organ: 0.063
Corr: 0.913***
>1 organ: 0.959***
1 organ: 0.304***
Corr: 0.887***
>1 organ: 0.946***
1 organ: 0.197***
Corr: 0.895***
>1 organ: 0.971***
1 organ: 0.199***
Corr: 0.875***
>1 organ: 0.957***
1 organ: 0.183***
Corr: 0.894***
>1 organ: 0.971***
1 organ: 0.178***
SB scores
SB scores

Fig. S2.

Correlation across organs of the magnitude and direction of sex bias for genes sex-biased in one organ and genes sex-biased in multiple organs in all species. The numerical value of the sex-bias score indicates magnitude of the sex bias, the sign indicates direction of the bias (negative means female bias, positive means male bias). Numbers in grey represent global Pearson's correlation coefficients for all sex-biased genes; numbers in red represent Pearson's correlation coefficients for genes sex-biased in more than 1 organ; numbers in blue represent Pearson's correlation coefficients for genes sex-biased in only 1 organ. ***, ** and * mean $P < 0.001$, $P < 0.01$ and $P < 0.05$, respectively.

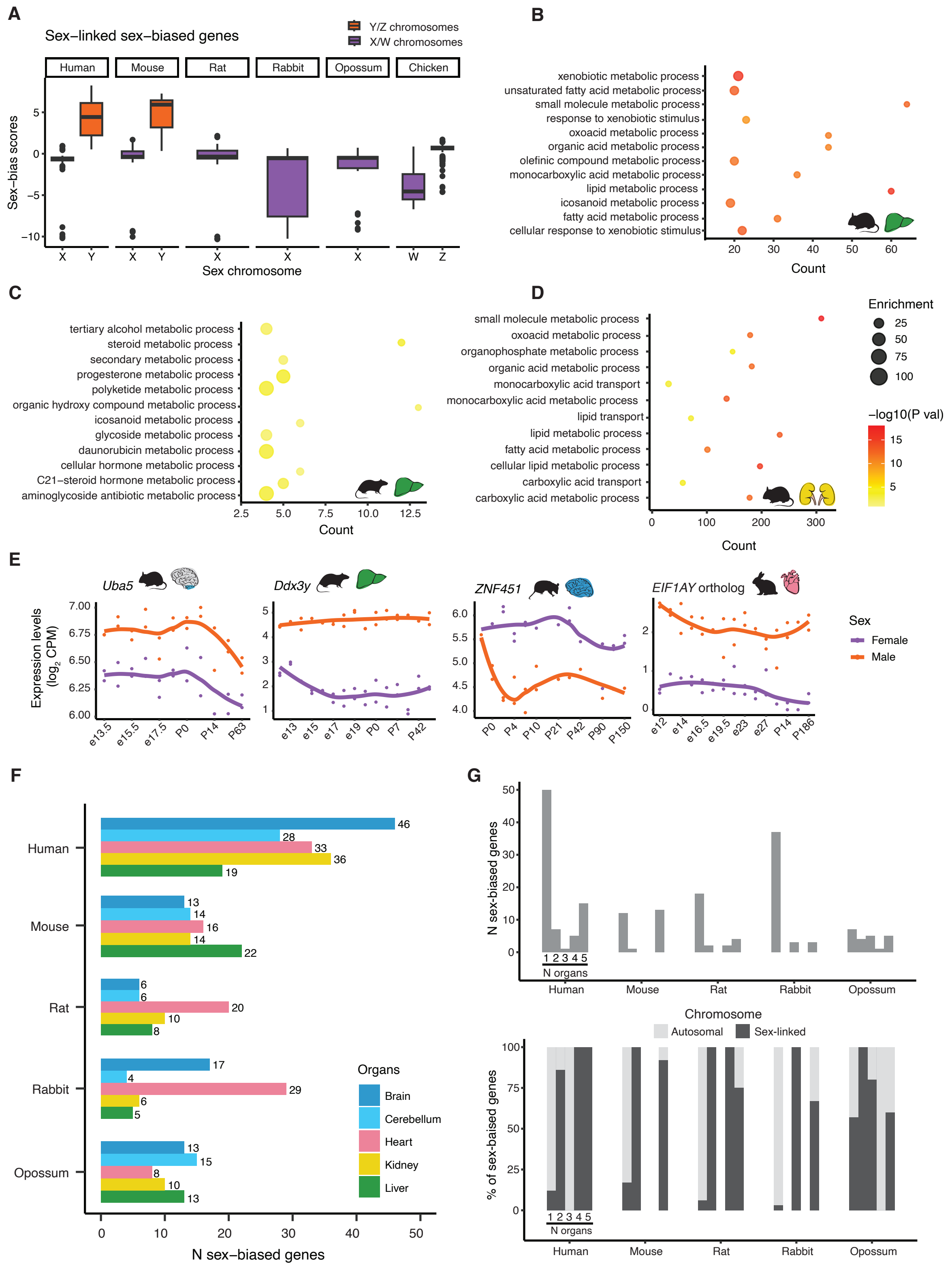

Fig. S3.

(A) Sex-bias scores of sex-linked sex-biased genes in each species. **(B-D)** Enriched biological processes among genes that become sex-biased after sexual maturity in mouse **(B)** and rat **(C)**

liver and mouse kidney **(D)** (n = 231 in mouse liver, n = 163 in rat liver and n=1876 in mouse kidney; Benjamini–Hochberg-adjusted $P < 0.05$, hypergeometric test). **(E)** Examples of autosomal always sex-biased genes in different species and organs. CPM = counts per million. **(F)** Number of sex-biased genes per organ and species that start differing between the sexes before or around birth and that are still sex-biased in adults. **(G)** Number of sex-biased genes (from **(F)**) and chromosomal location as a function of the number of organs where genes are sex-biased.

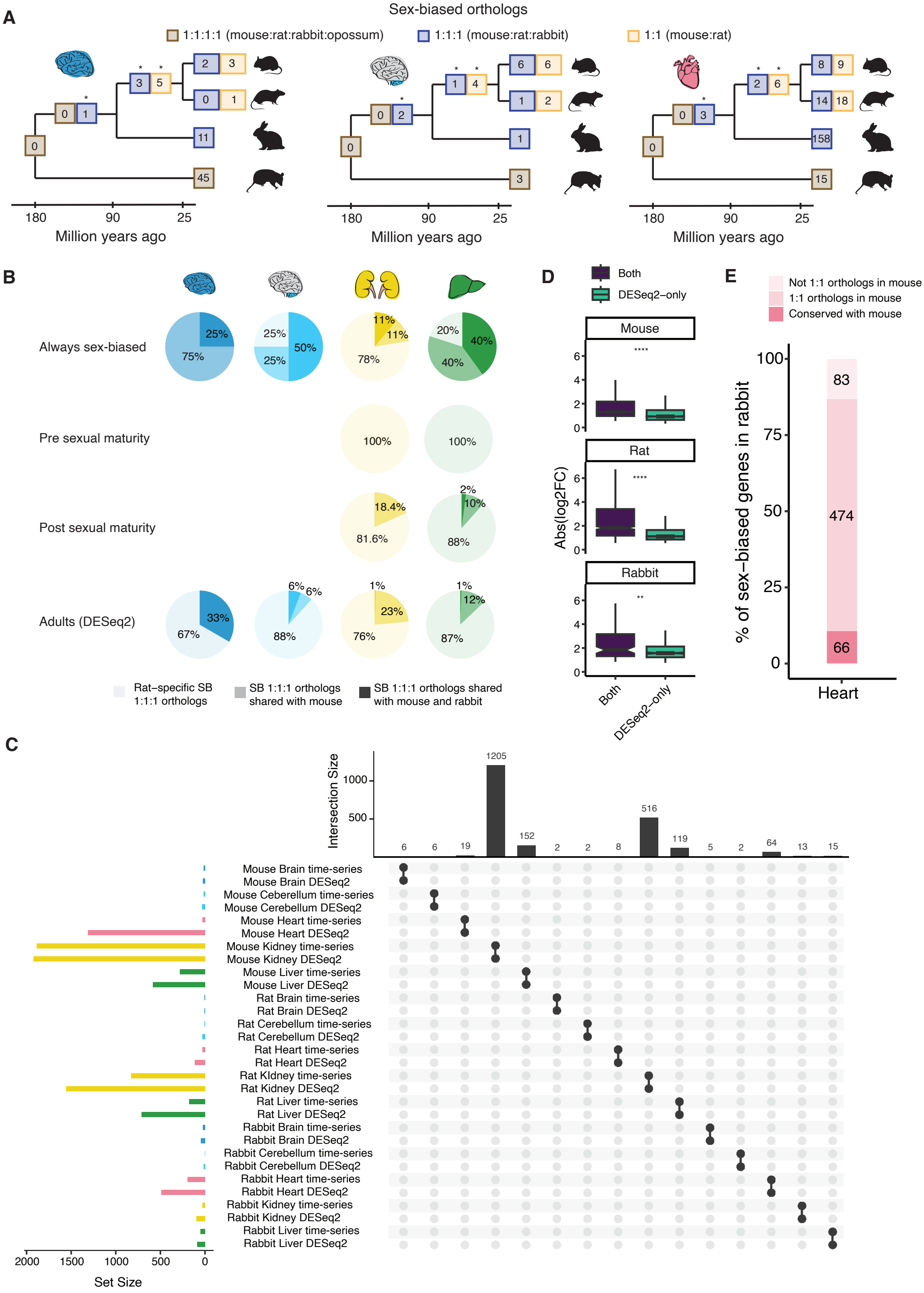
A
Sex-biased orthologs
1:1:1:1 (mouse:rat:rabbit:opossum)
1:1:1 (mouse:rat:rabbit)
1:1 (mouse:rat)
Million years ago
B
Always sex-biased
Pre sexual maturity
Post sexual maturity
Adults (DESeq2)
Rat–specific SB 1:1:1 orthologs
SB 1:1:1 orthologs shared with mouse
SB 1:1:1 orthologs shared with mouse and rabbit
C
Intersection Size
Set Size
Mouse Brain time-series
Mouse Brain DESeq2
Mouse Ceberellum time-series
Mouse Cerebellum DESeq2
Mouse Heart time-series
Mouse Heart DESeq2
Mouse Kidney time-series
Mouse Kidney DESeq2
Mouse Liver time-series
Mouse Liver DESeq2
Rat Brain time-series
Rat Brain DESeq2
Rat Cerebellum time-series
Rat Cerebellum DESeq2
Rat Heart time-series
Rat Heart DESeq2
Rat KIdney time-series
Rat Kidney DESeq2
Rat Liver time-series
Rat Liver DESeq2
Rabbit Brain time-series
Rabbit Brain DESeq2
Rabbit Cerebellum time-series
Rabbit Cerebellum DESeq2
Rabbit Heart time-series
Rabbit Heart DESeq2
Rabbit Kidney time-series
Rabbit Kidney DESeq2
Rabbit Liver time-series
Rabbit Liver DESeq2
D
Both
DESeq2–only
Mouse
Rat
Rabbit
Abs(log2FC)
E
Not 1:1 orthologs in mouse
1:1 orthologs in mouse
Conserved with mouse
% of sex–biased genes in rabbit
Heart

Fig. S4.

(A) Phylogeny showing the number of sex-biased orthologs in the brain, cerebellum and heart across mammals. * means Benjamini–Hochberg-adjusted $P < 0.05$, permutation test. The different numbers reflect the different sets of 1:1 orthologs used. For example, the set of 1:1 (mouse:rat) orthologs includes all 1:1:1 (mouse:rat:rabbit) orthologs plus genes that are only 1:1 orthologs between mouse and rat. **(B)** Percentage of sex-biased 1:1:1 orthologs in the rat brain, cerebellum, kidney and liver that are only sex-biased in rat, sex-biased in rat and mouse or sex-biased in rat, mouse and rabbit, depending on the onset of sex-biased expression. **(C)** Comparison of sex-biased genes detected with the time series approach and with classical differential expression analysis (using DESeq2) in adult samples for each organ in mouse, rat and rabbit. The number of genes detected with each method is depicted in the barplot on the left. The overlaps between both methods are depicted on the barplot on top. **(D)** Distribution of log2 fold-changes of sex-biased genes detected only with DESeq2 compared to the distribution of log2 fold-changes of sex-biased genes detected by both DESeq2 and the time series approach (**** $P < 0.0001$, two-sided Wilcoxon rank-sum test). **(E)** Number and percentage of sex-biased genes in rabbit heart that are either also sex-biased in mouse, have a 1:1 ortholog in mouse or do not have a 1:1 ortholog in mouse.

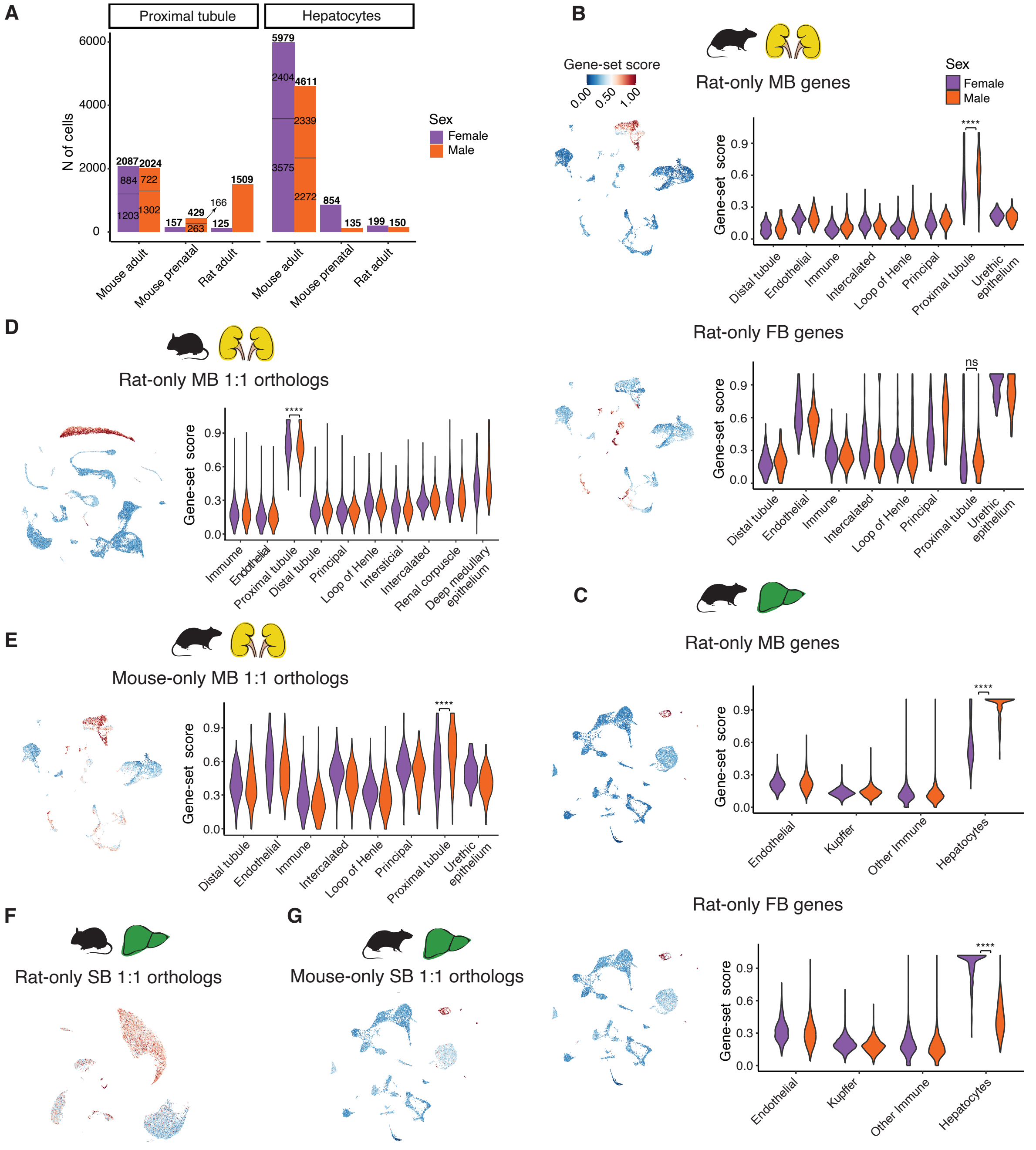

Fig. S5

(A) Number of cells/nuclei belonging to each sex per dataset. Data for number of nuclei/cells per biological replicate is indicated inside the barplot when reported by the authors. **(B, C)** UMAPs and violin plots illustrating expression of rat-only sex-biased genes in adult rat kidney **(B)** and liver **(C)** (**** means Benjamini–Hochberg-adjusted $P < 0.0001$, ns means not significant, two-sided Wilcoxon rank-sum test). **(D)** UMAP and violin plot illustrating expression of rat-only male-biased 1:1 orthologs in adult mouse kidney (**** means Benjamini–Hochberg-adjusted $P < 0.0001$, two-sided Wilcoxon rank-sum test). **(E)** UMAP and violin plot illustrating expression

of mouse-only male-biased 1:1 orthologs in adult rat kidney (**** means Benjamini–Hochberg-adjusted $P < 0.0001$, two-sided Wilcoxon rank-sum test). **(F)** UMAP illustrating expression of rat-only sex-biased 1:1 orthologs in adult mouse liver. **(G)** UMAP illustrating expression of mouse-only sex-biased 1:1 orthologs in adult rat liver.

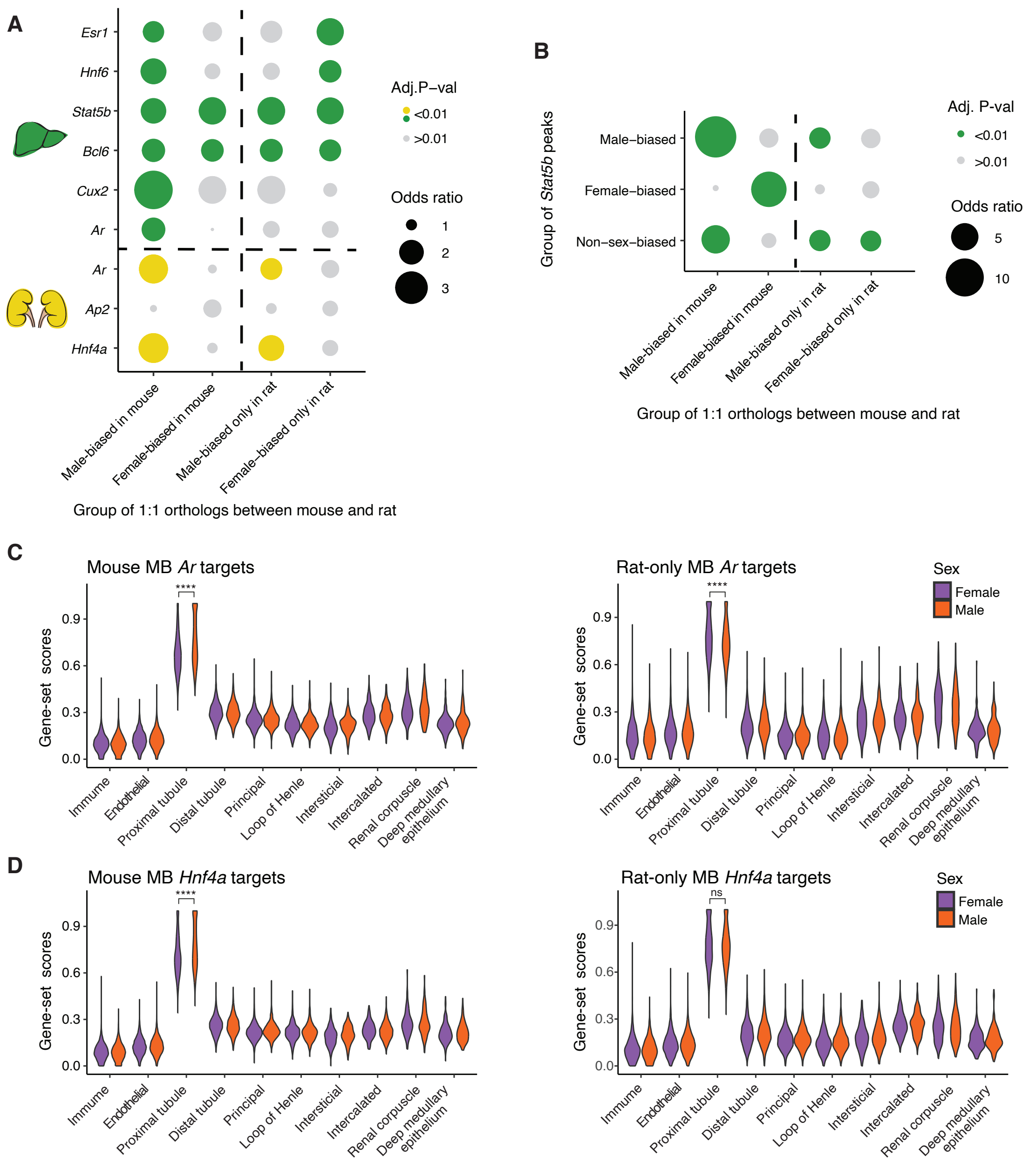

Fig. S6

(A) Enrichment of different groups of mouse 1:1 orthologs (those male- or female-biased in mouse and those only male-biased or female-biased in rat) for genes regulated by hormone-responsive or sex-biased transcription factors in mouse kidney and liver. **(B)** Enrichment of different groups of mouse 1:1 orthologs (those male- or female-biased in mouse and those only male-biased or female-biased in rat) for *Stat5b* peaks that show differences or not between the sexes. **(C)** Violin plots illustrating expression of mouse male-biased *Ar* targets in adult mouse kidney (**** means Benjamini–Hochberg-adjusted $P < 0.0001$, two-sided Wilcoxon rank-sum test) and of mouse 1:1 orthologs of *Ar* targets that are only male-biased in rat (but not in mouse) in adult mouse kidney (**** means Benjamini–Hochberg-adjusted $P < 0.0001$, two-sided Wilcoxon rank-sum test). **(D)** Violin plots illustrating expression of mouse male-biased *Hnf4a*

targets in adult mouse kidney (**** means Benjamini–Hochberg-adjusted $P < 0.0001$, two-sided Wilcoxon rank-sum test) and of mouse 1:1 orthologs of *Hnf4a* targets that are only male-biased in rat (but not in mouse) in adult mouse kidney (ns means not significant, two-sided Wilcoxon rank-sum test).

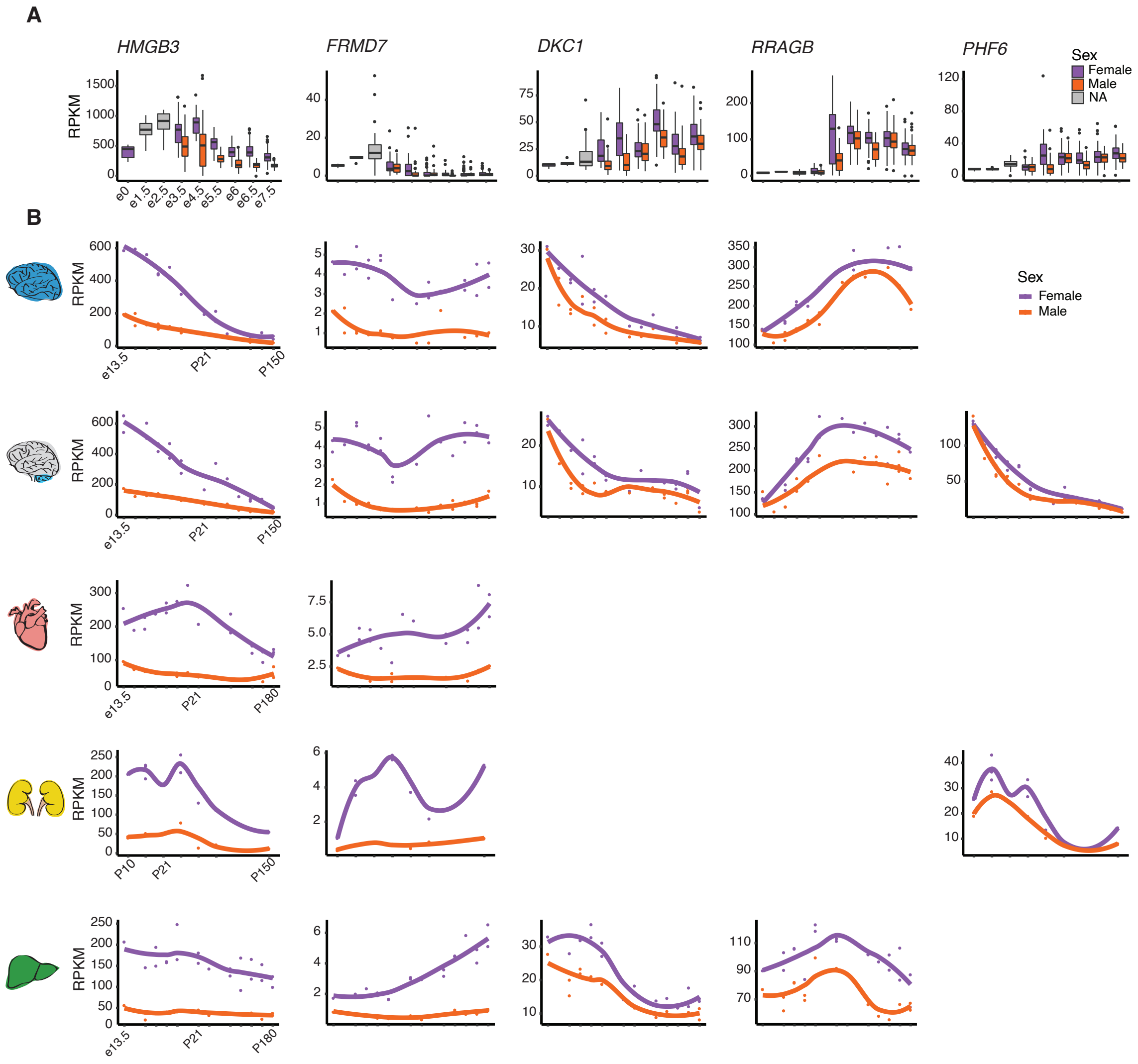

Fig. S7

(A) Gene expression time-courses of some of the always sex-biased X-linked opossum genes (*HMGB3*, *FRMD7*, *DKC1*, *RRAGB* and *PHF6*) in early embryonic stages (e0 to e7.5) of opossum (data from *(113)*). RPKM= Reads Per Kilobase per Million. **(B)** Gene expression time-courses of *HMGB3*, *FRMD7*, *DKC1*, *RRAGB* and *PHF6* in the organs where they are sex-biased in this study. RPKM= Reads Per Kilobase per Million.

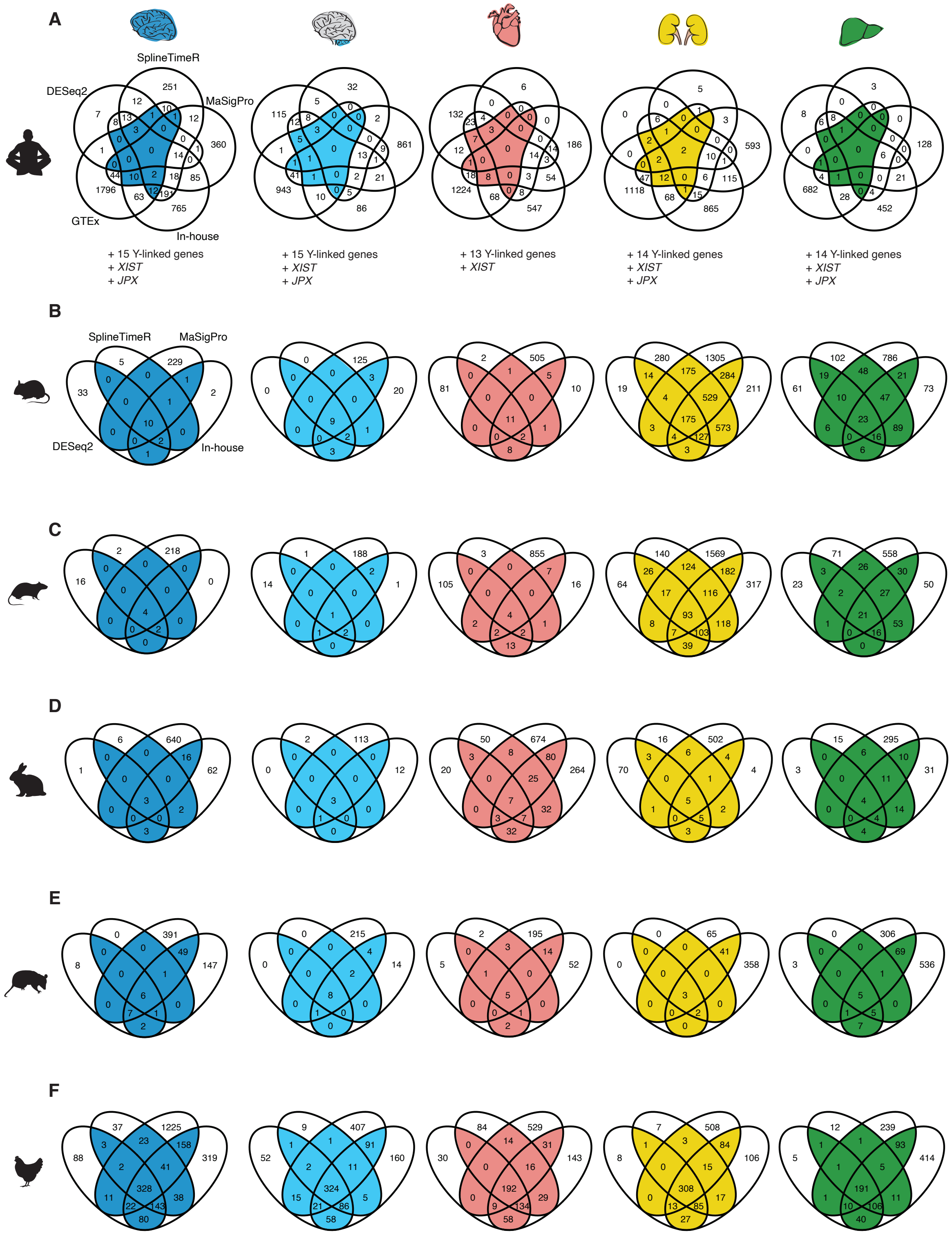

Fig. S8

(A) Venn diagrams of genes classified as sex-biased by SplineTimeR, MaSigPro, DESeq2 for time-series data, our own pipeline (in-house) and Oliva et al. *(9)* in each organ in humans. Manually added genes are specified below. Colored area indicates the overlaps considered for

the final set of sex-biased genes. **(B)** Venn diagrams of genes classified as sex-biased by SplineTimeR, MaSigPro, DESeq2 and our own pipeline in each organ in mouse, rat **(C)**, rabbit **(D)**, opossum **(E)** and chicken **(F)**. Colored area indicates the overlaps considered for the final set of sex-biased genes.

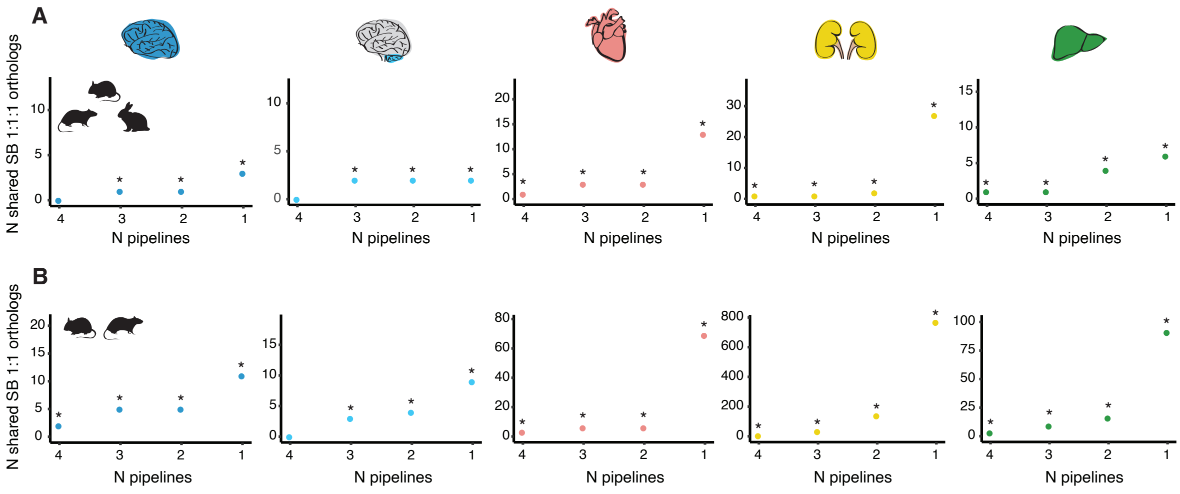

Fig. S9

(A) Number of shared sex-biased 1:1:1 orthologs in each organ across three eutherian species (mouse, rat, rabbit) as a function of the number of pipelines used for calling sex-biased genes (n=13387 1:1:1 orthologs across mouse, rat and rabbit). * means Benjamini–Hochberg-adjusted $P < 0.05$, permutation test. **(B)** Number of shared sex-biased 1:1 orthologs in each organ across two rodent species (mouse, rat) as a function of the number of pipelines used for calling sex-biased genes (n=16606 1:1 orthologs across mouse and rat). * means Benjamini–Hochberg-adjusted $P < 0.05$, permutation test.

Excel file with Tables S1 to S16 can be accessed in the following link:
https://www.dropbox.com/s/smdewbzuvh2ugza/New_Supplementary_tables_1_16.xlsx?dl=0

Table S1.
Time series for each organ and species (including number of replicates per sex).

<Excel file >

Table S2.
Sex-biased mouse genes and onset classification.

<Excel file >

Table S3.
Sex-biased rat genes and onset classification.

<Excel file >

Table S4.
Sex-biased rabbit genes and onset classification.

<Excel file >

Table S5.
Sex-biased opossum genes and onset classification.

<Excel file >

Table S6.
Sex-biased chicken genes and onset classification.

<Excel file >

Table S7.
Sex-biased human genes.

<Excel file >

Table S8.
Pipelines' results for human genes in all organs.

<Excel file >

Table S9.
Pipelines' results for mouse genes in all organs.

<Excel file >

Table S10.
Pipelines' results for rat genes in all organs.

<Excel file >

Table S11.
Pipelines' results for rabbit genes in all organs.

<Excel file >

Table S12.
Pipelines' results for opossum genes in all organs.

<Excel file >

Table S13.
Pipelines' results for chicken genes in all organs.

<Excel file >

Table S14.
Sex-biased phenotypes for early sex-biased mouse genes.

<Excel file >

Table S15.
Conserved sex-biased genes in mouse, rat and rabbit.

<Excel file >

Table S16.
List of X-linked genes in opossum that are always sex-biased in at least 2 organs.

<Excel file >
